# Synergistic antibacterial effects of ultrasound combined nanoparticles encapsulated with cellulase and levofloxacin on *Bacillus Calmette-Guérin* biofilms

**DOI:** 10.3389/fmicb.2023.1108064

**Published:** 2023-03-01

**Authors:** Zhifei Zhang, Yuqing Zhang, Min Yang, Can Hu, Hongjian Liao, Dairong Li, Yonghong Du

**Affiliations:** ^1^State Key Laboratory of Ultrasound in Medicine and Engineering, College of Biomedical Engineering, Chongqing Medical University, Chongqing, China; ^2^Chongqing Key Laboratory of Biomedical Engineering, Chongqing Medical University, Chongqing, China; ^3^Department of Respiratory and Critical Care Medicine, The First Affiliated Hospital of Chongqing Medical University, Chongqing, China

**Keywords:** ultrasound, *Bacille Calmette-Guerin* biofilms, nanoparticles, cellulase, levofloxacin

## Abstract

Tuberculosis is a chronic infectious disease, the treatment of which is challenging due to the formation of cellulose-containing biofilms by *Mycobacterium tuberculosis* (MTB). Herein, a composite nanoparticle loaded with cellulase (CL) and levofloxacin (LEV) (CL@LEV-NPs) was fabricated and then combined with ultrasound (US) irradiation to promote chemotherapy and sonodynamic antimicrobial effects on *Bacillus Calmette-Guérin* bacteria (BCG, a mode of MTB) biofilms. The CL@LEV-NPs containing polylactic acid-glycolic acid (PLGA) as the shell and CL and LEV as the core were encapsulated *via* double ultrasonic emulsification. The synthesized CL@LEV-NPs were uniformly round with an average diameter of 196.2 ± 2.89 nm, and the zeta potential of −14.96 ± 5.35 mV, displaying high biosafety and sonodynamic properties. Then, BCG biofilms were treated with ultrasound and CL@LEV-NPs separately or synergistically *in vivo* and *in vitro*. We found that ultrasound significantly promoted biofilms permeability and activated CL@LEV-NPs to generate large amounts of reactive oxygen species (ROS) in biofilms. The combined treatment of CL@LEV-NPs and US exhibited excellent anti-biofilm effects, as shown by significant reduction of biofilm biomass value and viability, destruction of biofilm architecture *in vitro*, elimination of biofilms from subcutaneous implant, and remission of local inflammation *in vivo*. Our study suggested that US combined with composite drug-loaded nanoparticles would be a novel non-invasive, safe, and effective treatment modality for the elimination of biofilm-associated infections caused by MTB.

## 1. Introduction

Tuberculosis (TB) is the second most common chronic infectious disease caused by *Mycobacterium tuberculosis* (MTB), with approximately 10.6 million newly diagnosed cases and about 1.6 million MTB-related deaths worldwide in 2021 (Getnet et al., 2021; Kasaeva, 2022). The current clinical treatment of TB is generally a combination of four first-line anti-tuberculosis drugs for 6 to 9 months, even lasting for 24 months in about 82% patients with multidrug-resistant TB (MDR-TB; Floyd et al., 2018). The long treatment time leads to poor patient compliance and poor treatment outcomes. Therefore, we urgently need to address the causes of MTB drug resistance and seek for new strategies to shorten the current TB treatment duration.

The National Institutes of Health (NIH) agency reports that 65% of microbial diseases and more than 80% of chronic infections are associated with bacterial biofilms, including cystic fibrosis, sinusitis, non-healing wounds, and implanted catheters related infection (Wolfmeier et al., 2018; Hou et al., 2019; Hu et al., 2020). Biofilms associated with antibiotic resistance have been reported to exist in MTB, and the extracellular material in these biofilms is mainly composed of polysaccharides, of which cellulose is a key component (Trivedi et al., 2016). In fact, biofilms are mainly the extracellular polymeric substances (EPS), composed of eDNA, polysaccharides, proteins, and lipids, which form a natural barrier to resist host immune responses and drug attacks (Flemming et al., 2007; Alipour et al., 2009; Limoli et al., 2015; Flemming, 2016). Thus, biofilm formation is more conducive for bacteria to develop resistance to drugs.

Sonodynamic antimicrobial chemotherapy (SACT), on the basis of sonodynamic therapy (SDT), has been established and developed as a promising alternative for microbial inactivation. SACT is a combination of ultrasound and chemotherapeutic agents termed sonosensitizers to produce highly oxidative active reactive oxygen species and ultimately achieve treatment through the removal of bacterial biofilm infections (Yang et al., 2019; Lai et al., 2022). Compared with conventional treatment modalities, SACT has superior penetration ability and high target specificity, and can overcome resistance conferred by the local microenvironment. Levofloxacin (LEV) is a highly effective broad-spectrum antibiotic used clinically and a second-line anti-TB drug (Koh et al., 2013). Besides, LEV has been demonstrated to act as a potential sonosensitizer drug for SDT in previous studies, which provides the possibility for its combination with ultrasound to achieve highly effective acoustic dynamic antibacterial therapy (Hytonen et al., 1994; Liu et al., 2010, 2011; Chen et al., 2014; Xie et al., 2020).

Cellulose is an important component in the extracellular matrix of the biofilm pathogenic bacteria, including MTB formed biofilms, so efficient removal of cellulose from the biofilm is considered as one of the key anti-biofilm treatment strategies. Cellulases, a glycoside hydrolase, were discovered a long time ago that specifically exert its role in disrupting bacterial biofilms by breaking down the β-1,4 bond in polysaccharides (Van Wyk et al., 2017) (cellulose). A previous study carried out elsewhere has reported that cellulase in combination with antibiotics therapy can be effective to inhibit *Pseudomonas aeruginosa* biofilms formation and promote biofilms clearance (Kamali et al., 2021), which is necessary to disperse the bacteria to the planktonic state is necessary (Losada et al., 1986; Hytonen et al., 1994; Wang et al., 2021). However, CL is an exogenous mammalian protein and prolonged exposure to it can cause several adverse reactions such as asthma, pharyngeal edema, and rhinitis (Xie et al., 2020; Li et al., 2021). Therefore, there is a need to develop a novel CL treatment strategy with higher drug efficacy and lower drug toxicity.

Nanodrug delivery systems have aroused great attention in the treatment of antitumor and biofilm-associated infections because of their advantages of improved drug efficacy, reduced drug dosage, and reduced toxic side effects through the slow release of drugs in the target tissue (Yang et al., 2019; Sun et al., 2020). Polylactic acid-glycolic acid (PLGA) is one of the most commonly used biodegradable nanomaterials and has been approved by the Food and Drug Administration (FDA) for use in humans as a nanodrug delivery system with good biodegradability and biocompatibility (Naumoff, 2011). Besides, the presence of nanoparticles (NPs) can be used as exogenous cavitation nuclei to reduce the cavitation threshold and amplify the cavitation effect, which can facilitate the activation of the sonosensitizer to enhance the bactericidal effect of SDT (Salgaonkar et al., 2009; Suk et al., 2016). *Bacille Calmette-Guerin* (BCG) is one of the model strains of MTB bacterium grown with similarities of genomic as well as cell wall structure and composition to MTB. Therefore, BCG is an ideal substitute strain for anti-MTB experimental studies (Maurya et al., 2020).

**Figure 1:**
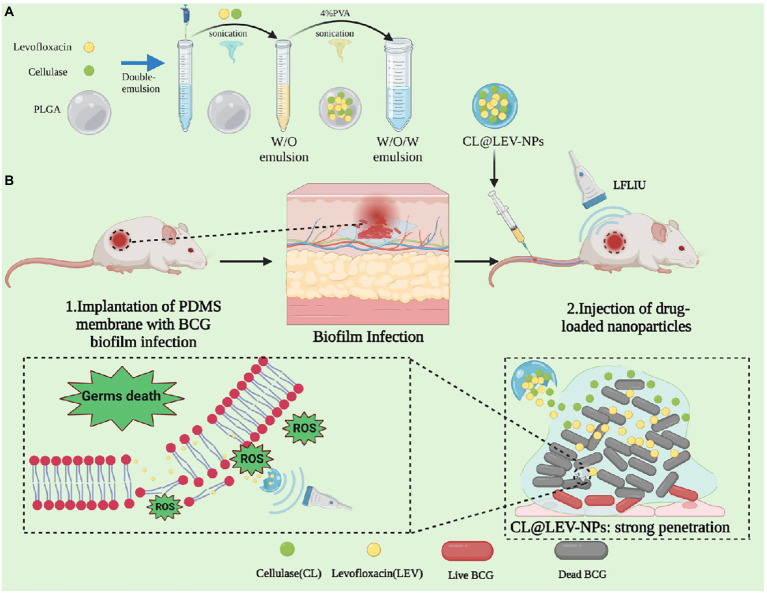
Schematic diagram of biofilm dispase combined with antibiotic composite nanoparticles for sonodynamic antimicrobial chemotherapy of BCG biofilm infections.

In this study, we have successfully fabricated the composite nanoparticles based on PLGA polymerized organic material loaded with (CL@LEV-NPs) and further combined them with ultrasound to achieve the acoustic dynamic antibacterial effect of against BCG bacterial biofilms *in vitro* and *in vivo*. US was utilized to trigger the co-release of CL and LEV from CL@LEV-NPs. CL promoted the disassembly of BCG biofilms and deprived the protection of the internal bacteria, while simultaneously LEV eliminated BCG biofilms and killed bacteria inside the biofilms. The schematic diagram of biofilms dispase combined with antibiotic composite nanoparticles for SACT against the BCG biofilm infections is seen in Figure 1.

## 2. Materials and methods

### 2.1. Materials

PLGA-COOH (MW 15000, Jinan Daigang Biotechnology Co., LTD.), Chloroform (CHCl_3_, MW 119.38), Isopropyl alcohol (MW 60.10, Chongqing Chuandong Co., LTD.), Polyvinyl alcohol (PVA,MW30000-70000), Middlebrook’s 7H9 broth medium, and Oleic Acid-Albumin-Dextrose-Catalase (OADC) were purchased from BD biosciences (New York, USA), 1,1′-dioctadecyl-3,3,3′,3′-tetramethylindo carbocyanine perchlorate (DiI) red fluorescence probe, Tween-80, glycerol, cellulase (CL), Calcofluor White Stain (CW), Methyl tetrazolium salt (XTT), and menadione were purchased from Sigma. LIVE/ DEAD (SYTO 9 / PI) BacLight Bacterial Viability Kit (L7012) was purchased from Thermo Fisher Technologies. Levofloxacin (LEV), crystal violet, and citrate buffer were purchased from Beijing Solebao Technology Co., Ltd. 2,7-dichlorodihydrofluorescein diacetate (DCFH-DA) was bought from Beyotime Biotechnology Co., Ltd. (Shanghai, China). Singlet Oxygen Sensor Green (SOSG) was bought from Thermo Fisher Scientific. ELISA kits for IFN-γ, IL-1α, IL-6, and TNF-α were obtained from Jingmei Biotechnology (Jiangsu, China).

### 2.2. Bacteria culture and biofilms formation establishment

BCG was originally obtained from Chengdu Institute of Biological Products, Chengdu, China. BCG was grown in Middlebrook 7H9 broth supplemented with 10% Middlebrook OADC, 0.2% glycerol, and 0.5% Tween-80 at 37°C and shaken (shaker speed, 200 r/min) until logarithmic growth period (OD_600_ = 1.0). Then, the culture was centrifuged at 4,000 r/min for 10 min and the bottom bacteria were re-suspended in Middlebrook 7H9 Broth without Tween-80. After that, the bacteria were dropped them into 35 mm Petri dish (2 mL of bacterial solution per dish) and 96-well plates (200 μL of bacterial solution per well), respectively, then were placed in a 37°C incubator in static culture to allow the formation of a mature biofilms after about 10 days, which were finally confirmed by crystal violet staining and XTT assay (Supplementary Figures S1D,E).

### 2.3. Animal species

The animals used in the experiments were SPF-grade BALB/c female mice (4–6 weeks old, 18–22 g), which were provided by the Laboratory Animal Center of Chongqing Medical University. All experimental animals were housed in monomer independent air cages and given standard diet. Animal study was performed in accordance with the Regulations for the Management of Laboratory Animals and the Guidelines of the China Laboratory Animal Guideline for Ethical Review of Animal Welfare (2022154), which was approved by the Animal Ethics Committee of Chongqing Medical University.

### 2.4. Synthesis and characterization of CL@LEV-PLGA nanoparticles

Cellulase and levofloxacin nanoparticles (CL@LEV-NPs) were prepared by a double emulsion method in the following steps. First, 40 mg PLGA-COOH was dissolved in 2 mL trichloromethane and mixed with 300 μL aqueous phase (100 μL CL, 0.512 mg/mL and 200 μL LEV, 5 mg/mL). The mixture was emulsified in an ice bath by using an ultrasonic probe (XL2020 Acoustic Vibrograph, USA) with the power of 150 W (70% amplitude) for 1 min. Then, 4 mL 4% polyvinyl alcohol (PVA) was added into the initial emulsion for second emulsification for 2.5 min to form a double emulsion. Subsequently, the obtained emulsion was added into 6 mL of 2% isopropanol and magnetically stirred for 3–6 h (80–90 r/min) to completely volatilize the organic solvent. Sterile water-loaded nanoparticles (Blank-NPs), cellulase-loaded nanoparticles (CL-NPs), and levofloxacin-loaded (LEV-NPs) and DiI-loaded nanoparticles (DiI-NPs) were produced in the similar manner. All the obtained nanoparticles were stored by freeze-drying.

The morphology and distribution of prepared nanoparticles were evaluated by a scanning electron microscope (SEM, Hitachi SU8010, Japan) and transmission electron microscope (TEM, Hitachi H-7600, Japan). The particle size and Zeta potential of the nanoparticles were detected using a Malvern laser granularity instrument (Zeta SIZER 3000HS, America). The CL@LEV-NPs were dispersed in PBS and the change in particle size was measured over 7 days to evaluate the stability of the nanoparticles. The loading efficiency (DL) and encapsulation efficiency (EE) of drug CL and LEV in CL@LEV-NPs were calculated by the following formulas:

DL% = (drug content in nanoparticles/loaded nanoparticle quality) × 100%;

EE% = (drug content in nanoparticles/total drug input) × 100%.

#### 2.4.1. *In vitro* drug release experiments with US stimulation

The *in vitro* drug release profiles from nanoparticles were conducted using the dialysis method. CL@LEV-NPs (20 mg) were re-suspended in 2 mL PBS and irradiated with ultrasound at intensity of 0.34 W/cm^2^ with irradiation for 10 min. Then, the nanoparticles dispersion was transferred into a dialysis bag (MWCO: 12 kDa, Spectrum Laboratories, CA, USA). The dialysis bag was placed into a beaker containing 25 mL PBS and stirred by a magnetic stirrer, with a speed of 120 r/min at 37°C to homogenize the solution in the beaker. At pre-determined time points (0, 1, 2, 4, 8, 12, 24, 48, and 72 h), the CL and LEV concentrations of each sample were detected the by using the UV–vis spectrophotometer with absorption values at 303 nm and 281 nm, respectively. The cumulative LEV and CL releases were calculated using the following formula:

Cumulative release of LEV = (amount of LEV in PBS solution/total LEV in CL@LEV-NPs) × 100%;

Cumulative release of CL = (amount of CL in PBS solution/total CL in CL@LEV-NPs) × 100%.

#### 2.4.2. Detection of sonodynamic properties

Singlet oxygen sensor green (SOSG) was used as singlet oxygen detector to detect the production of reactive oxygen species (ROS; Martins et al., 2020). SOSG solution (5 μm, 0.5 mL) was added to LEV free drug solutions and the corresponding drug-loaded nanoparticle. Then, the solution was irradiated for 5 min with or without ultrasound (42 kHz, 0.34 W/cm^2^). The fluorescence intensity of the solution was measured on a fluorescence spectrophotometer with the wavelength of 504 nm excitation and 525 nm emission, and the generation of ROS after ultrasound irradiation was recorded.

### 2.5. Detection of antimicrobial susceptibility of LEV and CL to biofilms

#### 2.5.1. Antibacterial susceptibility testing of LEV

After resuscitation, BCG strain was inoculated in 7H9 broth medium and incubated in shaker at 37°C (200 r/min) until the OD_600nm_ of BCG solution was 1.0, then the concentration of the solution was adjusted to 10^7^ CFU/mL for backup (Gutiérrez-Larraínzar et al., 2013). Antimicrobial tests were performed to determine the MIC of planktonic cells for LEV according to the Clinical and Laboratory Standards Institute (CLSI) antimicrobial susceptibility test operation (Jean et al., 2021).

#### 2.5.2. Minimum biofilms eradication concentration assay of LEV

The antimicrobial susceptibility test of BCG biofilm was evaluated as described by Jean et al. (2021). After obtaining mature biofilms in 96-well plates, a 200 μL sample of each concentration (LEV, 0–2048 μg/mL) was added to a corresponding well, and plates were incubated overnight at 37°C. Wells that were not treated with LEV (7H9 medium without antibiotics was added) served as controls. After 24 h of warming, each well was gently washed three times with sterile PBS to remove residual antibiotics, and 200 μL of fresh Tween-80-free 7H9 medium was added to each well and further incubated at 37°C for 24, 48, and 60 h, allowing for bacteria that survived antibiotic exposure to grow without antibiotics and produce detectable turbidity. Subsequently, the OD of each well was measured at 590 nm, and the lowest antibiotic concentration that prevented bacterial regeneration in the treated biofilm was defined as the MBEC value (Losada et al., 1986).

#### 2.5.3. Inhibition of biofilms formation of cellulase

After obtaining mature BCG biofilms, the BCG biofilms were treated with different concentrations of CL (1.024, 0.512, 0.256, and 0.128 mg/mL). CL solution (200 μL) was added to each 96-well plate, and PBS or cellulase after high-temperature inactivation was added as the controls. After treatment, the plates were incubated in an incubator at 37°C for 24 h. The supernatant was gently aspirated, PBS was washed three times, and then 100 μL of 0.1% crystal violet solution was added to each well to assess the biomass of biofilms by crystal violet staining. The results were expressed as the percentage reduction in biofilm biomass compared to the control or HI groups (Heat-inactivated CL).

#### 2.5.4. Bactericidal activity of cellulase against planktonic bacteria

To verify whether cellulase has bactericidal activity against planktonic bacteria, a study was conducted on planktonic BCG. Briefly, different concentrations of CL (1.024, 0.512, 0.256, and 0.128 mg/mL) were added to 96-well plates containing BCG (OD_600_ = 1.0) for co-incubation, and wells of PBS-treated bacteria were used as controls. After 0, 24, and 48 h incubation at 37°C, the effect of the enzyme on the bacterial growth was determined by measuring the optical density (OD) of each culture in wells at 570 nm (Losada et al., 1986).

### 2.6. Penetration analysis of ultrasound mediated nanoparticles in BCG biofilms

**Figure 2:**
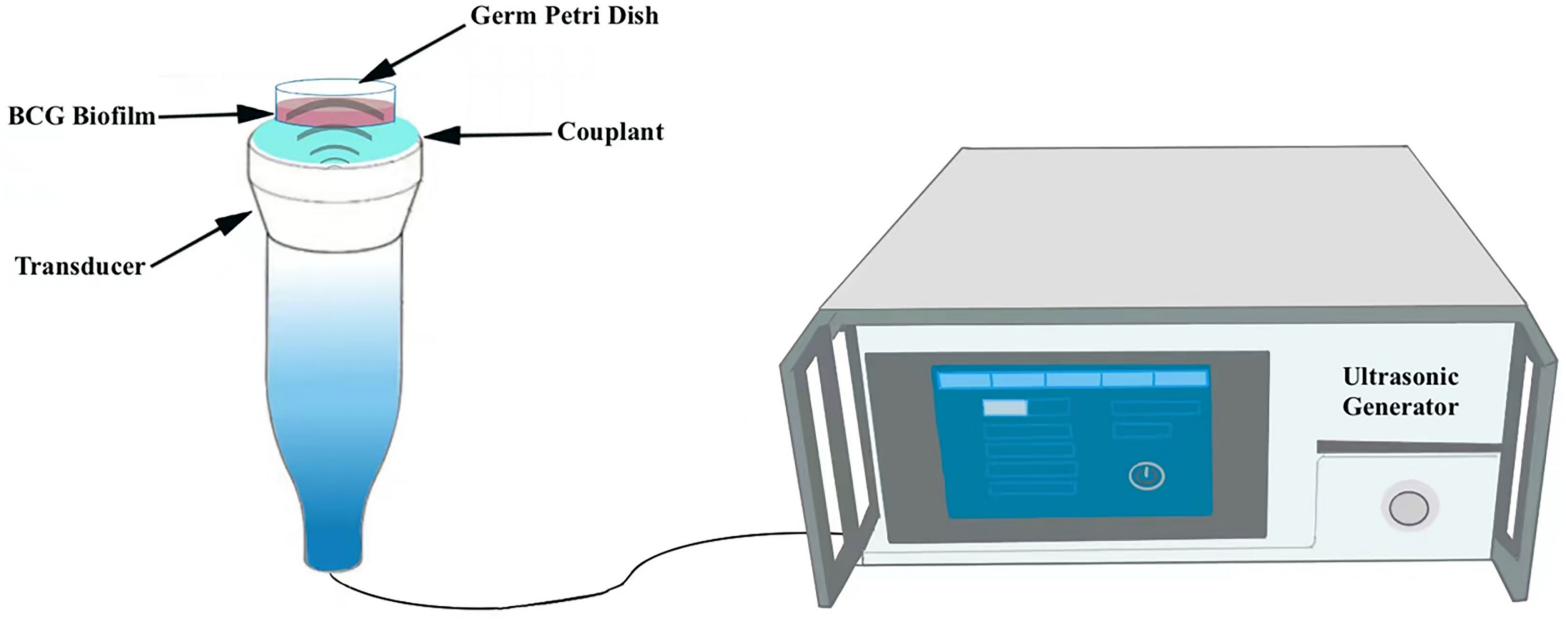
Schematic diagram of the ultrasound irradiation process. The germ culture dish is placed upright in the transducer.

DiI-NPs were used to evaluate the permeability of nanoparticles in BCG biofilms by confocal laser scanning microscopy (CLSM, A1R; Nikon, Tokyo, Japan) after ultrasonic irradiation. Briefly, 400 μL of DiI-NPs (red fluorescence) solution was added to the mature BCG biofilms developed on the cell culture plate and then the plate was placed directly above the transducer, followed by ultrasound irradiation as shown in Figure 2. The ultrasonic parameters used in this study were selected at a fixed frequency of 42 kHz and an intensity of 0.34 W/cm^2^ with irradiation for 10 min based on the result of percutaneous radiation safety testing (Supplementary Figure S3). After 12 h of incubation, the biofilms were washed three times with PBS and then stained with SYTO 9 for 30 min. Subsequently, the biofilms were washed with PBS to rinse SYTO 9 as well as non-infiltrating nanoparticles. Finally, the penetration of DiI-NPs into the biofilms was observed by CLSM with a scanning thickness of 3 μm for each layer, and the distribution of DiI-NPs at the bottom, middle, and top layer of the biofilm was compared. At the same time, the quantitative analysis of red fluorescence of DiI-NPs was performed using ImageJ software.

### 2.7. Anti-biofilm effect of ultrasound combined nanoparticles *in vitro*

**Figure 3:**
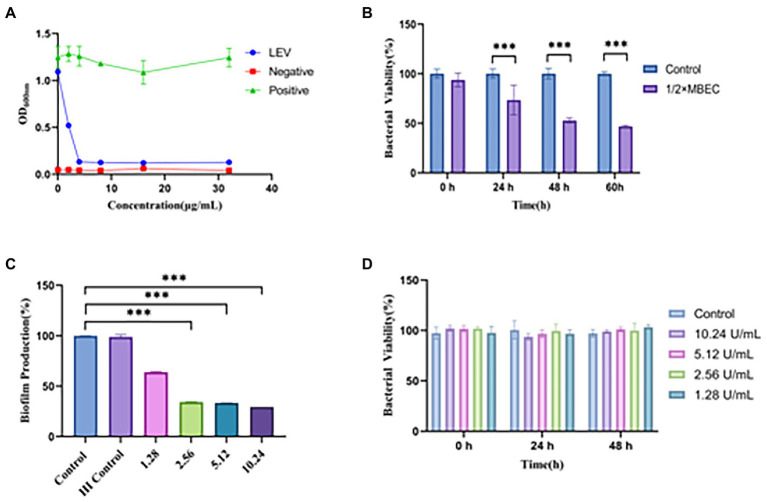
Antimicrobial susceptibility of LEV and CL to biofilm. **(A)** OD_600_ values of BCG solutes after treatment with different concentrations of LEV, Negative (adding only blank broth without bacterial solution), Positive (adding bacterial solution without drug solution); **(B)** Effect of different times of LEV action of 1/2 × MBEC on the activity of BCG biofilm; **(C)** Inhibition effect of different concentrations of CL on BCG biofilm; **(D)** Effect of different concentrations of CL on planktonic BCG ***p < 0.001; HI: Heat-inactivated; Percentage of bacterial viability = (*A*_treatment_ − *A*_blank_)/(*A*_control_ − *A*_blank_) × 100% (where, *A* = absorbance).

To further explore the anti-biofilm effect of ultrasound combined nanoparticles *in vitro*, mature biofilms were established in 35 mm Petri dishes and then treated with ultrasound and CL@LEV-NPs separately or synergistically and grouped as follows: control group (Control, only PBS without US and drug), ultrasound group (US), CL-loaded nanoparticles group (CL-NPs), LEV-loaded nanoparticles group (LEV-NPs), ultrasound combined CL-loaded nanoparticles group (US+CL-NPs), ultrasound combined LEV-loaded nanoparticles group (US+LEV-NPs), CL and LEV composite nanoparticles group (CL@LEV-NPs), and ultrasound combined CL and LEV composite nanoparticles group (US+CL@LEV-NPs). The final concentrations of CL and LEV in the corresponding drug-loaded nanoparticles were 0.512 mg/mL and 4 μg/mL (based on sensitivity testing, Figure 3), respectively. The ultrasound-related group was also irradiated with US at a frequency of 42 kHz and an intensity of 0.34 W/cm^2^ for 5 min. The ultrasonic irradiation method is the same as described above (Figure 2). The experiments were repeated independently three times.

#### 2.7.1. Evaluation of biofilms biomass

After 24 h of post-treatment reaction, the supernatant was gently washed three times with PBS, and 1 mL of 0.1% crystal violet solution was added to each dish for staining for 10 min, then the unbound crystal violet was washed away with PBS. After air drying at room temperature, 1 mL of 33% acetic acid solution was added for decolorizing, and the biofilm biomass of different groups was determined by measuring the absorbance at 570 nm using a microplate reader.

#### 2.7.2. Analysis of biofilms activity and structure by CLSM

SYTO 9/PI dyes were employed to perform a fluorescence-based biofilms live/dead observation. After the biofilms treatment with different methods, the biofilms were incubated with 100 μL fluorescent dye SYTO 9 for 30 min, gently washed three times in PBS, then 100 μL fluorescent dye PI was added to avoid light and incubated for another 5–10 min in the dark. Finally, the 3D reconstruction map and maximum area projection (200×) of BCG biofilm were visualized under CLSM.

#### 2.7.3. SEM observation of biofilms morphology and ultrastructure

Before the experiment, special coverslip from the electron microscopy room was placed in a 12 well plate. A mature BCG biofilm was established on this coverslip and treated by different ways. After that, it was washed with PBS for three times, and gently added 2.5% glutaraldehyde fixative for 4 h at 4°C. The damage effects of differently treated BCG biofilms on ultrastructural morphology were observed by SEM.

#### 2.7.4. ROS generation

*In Vitro* The level of intracellular ROS was detected using the redox-sensitive probe DCFH-DA. DCFH-DA itself is not fluorescent, but can react with ROS and convert to green fluorescent 2′,7-dichlorofluorescein (DCF), which is used as a probe to monitor intracellular ROS accumulation. After BCG biofilms were treated in different ways, medium containing DCFH-DA (1,000 μL, 1 μM) was added to the culture dish and co-cultured for 30 min to generate the steady green fluorescence signals (DCF) for observation by CLSM. Besides, the fluorescence intensity of DCF was quantitatified by flow cytometry (CytoFLEX, Beckman Coulter, Inc. CA, USA). Next, the dishes were rinsed thrice with sterile PBS to eliminate redundant DCFH-DA probes.

### 2.8. Establishment of subcutaneous BCG biofilms infection *in vivo*

The BCG biofilm implant infection model was established using polydimethylsiloxane (PDMS) slices as reported by previous literature (Naparstek et al., 2014). PDMS sections (7 × 4 mm) were completely immersed in 3 mL of 7H9 medium containing BCG (10^8^ CFU/mL) and incubated for 10 days (37 ± 0.5°C) to form biofilms on the surface of PDMS sections. In addition, methylprednisolone (5.4 mg/kg) was injected intramuscularly into the right buttock of BALB/c mice for three consecutive days prior to implantation to suppress immunity. After that, PDMS slices were surgically implanted into the back of the mice. PDMS slices infected by biofilms were sandwiched between the muscle and epidermis, separated from the muscle by the peritoneum. Three days after implantation, a portion of the tissue was taken and BCG biofilm infection was confirmed by acid fast staining (Supplementary Figure S4).

#### 2.8.1. Synergistic therapy with ultrasound and drug-loaded nanoparticles *in vivo* evaluation

After successful establishment of the subcutaneous BCG biofilm infection in mice, mice infected with BCG were randomly grouped (n = 5 in each group): (1) PBS alone (Control), (2) PBS with ultrasound (US), (3) CL-NPs alone (CL-NPs), (4) LEV-NPs alone (LEV-NPs), (5) CL@LEV-NPs alone (CL@LEV-NPs), (6) CL-NPs with ultrasound (US+CL-NPs), (7) LEV-NPs with ultrasound (US+LEV-NPs), and (8) CL@LEV-NPs with ultrasound (US+CL@LEV-NPs). For *in vivo* administration, mice received tail vein injections with a dose of 0.2 mL PBS or the suspension of different NPs (corresponding to LEV concentration of 5 mg/kg, CL concentration of 0.512 mg/mice) once every 2 days for 10 days. For the ultrasound-related groups, ultrasound treatment (42 kHz, 0.34 W/cm^2^) was performed once for 5 min at 24 h after each injection. The skin sample exposed to ultrasound with the parameters (the intensity of 0.34 W/cm^2^ for 5 min) showed no obvious changes in the skin surface (Supplementary Figure S3). At 2 days after treatment, PDMS slices and surrounding tissues were taken out from the mice for the bacterial count as previously reported (Tang et al., 2021; Zuo et al., 2021). Serum had been taken from the mice before they were sacrificed, and was used for cytokine, liver, and kidney function measurements. A hematoxylin and eosin (H&E) staining assay was utilized for observing the histological condition of skin tissues surrounding the implanted sites. Meanwhile, their organs (heart, liver, spleen, lung, and kidney) were carefully were carefully removed, embedded, sectioned, and processed for H&E staining.

Toxicity Assays *in Vitro* and *in Vivo* Safety is a prerequisite for the use of nanoparticles for *in vivo* experiments. We evaluated the effect of CL@LEV-NPs on erythrocytes *in vitro* by incubating different concentrations of CL@LEV-NPs with erythrocytes and observing the hemolysis. To further evaluate the *in vivo* safety of CL@LEV-NPs, peripheral blood was collected from BALB/c mice of different experimental groups at the end of *in vivo* treatment (on day 10 after injection) for blood biochemical analysis. The heart, liver, spleen, lung, and kidney of BALB/c mice were also collected for histopathological analysis.

### 2.9. ELISA quantification assay

ELISA kits (Jiangsu Jingmei Biotechnology Co., Ltd., China) were used in accordance with the manufacturer’s instruction. Mouse IFN-γ ELISA Kit, Mouse IL-1α ELISA Kit, Mouse IL-6 ELISA Kit, and Mouse TNF-α ELISA Kit were employed to measure the expression of immune factors in serum.

### 2.10. Statistical analysis

Statistical analysis was performed using GraphPad Prism version 9.0 for Windows (GraphPad Software; La Jolla, CA, United States). All data were expressed as mean ± standard deviation (SD). Significant differences among groups were analyzed using a one-way ANOVA and differences for individual groups were determined using Student’s *t*-test. The results were regarded as a significant difference when *p < 0.05. **p < 0.01, ***p < 0.001.

## 3. Results

### 3.1. Physical properties of the nanoparticles

**Figure 4:**
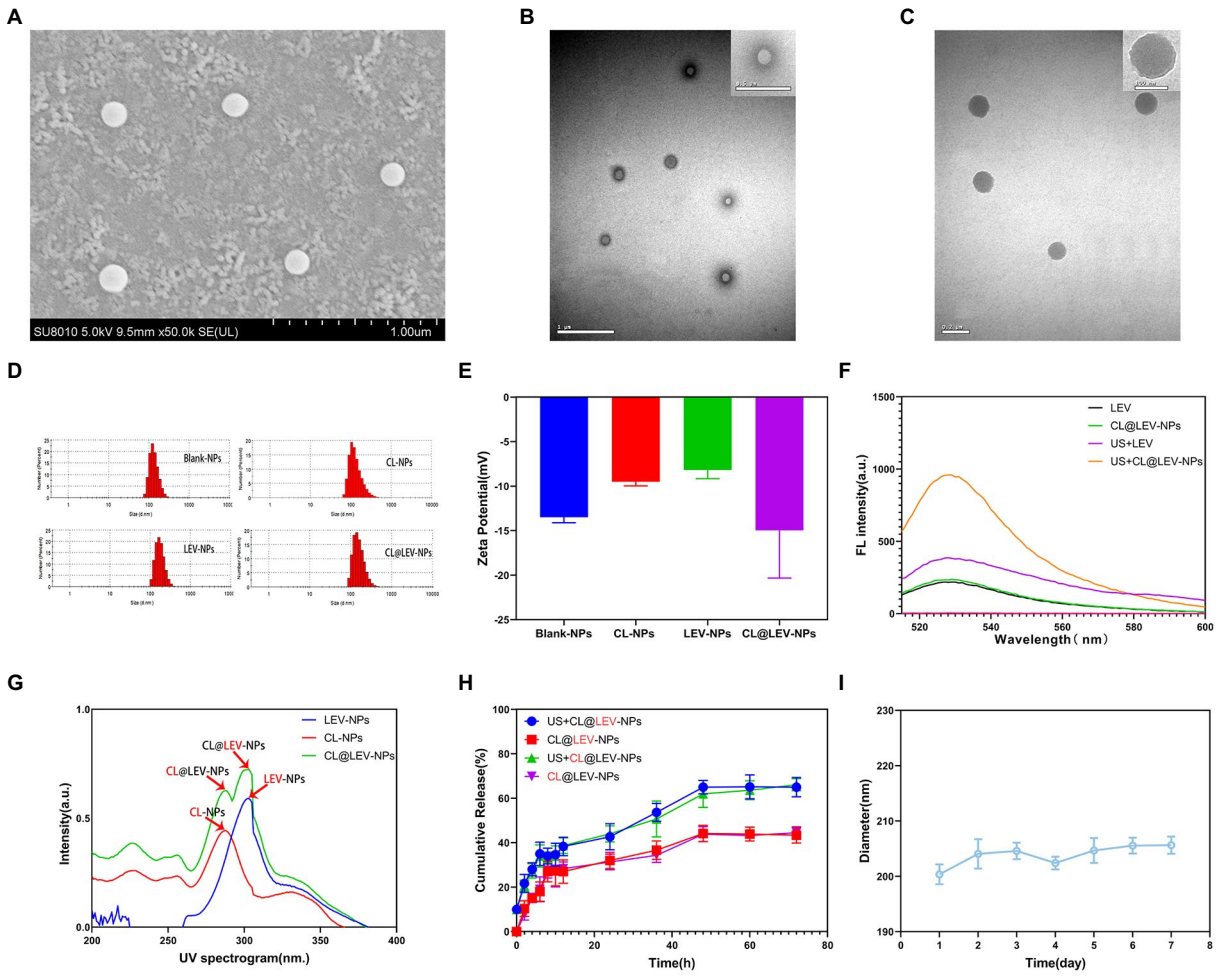
Basic properties of nanoparticles. **(A)** SEM observation of CL@LEV-NPs (SEM × 50 k); **(B)** TEM observation of Blank-NPs (TEM × 20 k OR ×40 k); **(C)** TEM observation of CL@LEV-NPs (TEM × 50 k OR ×120 k); **(D)** Particle size distribution of different nanoparticles; **(E)** Zeta potential of different nanoparticles; **(F)** SOSG detection of singlet oxygen of different drugs; **(G)** UV spectra of different drug-loaded nanoparticles; **(H)** Release profiles of two drugs from nanoparticles (CL@LEV-NPs) with or without ultrasound irradiation (0–72 h). **(I)** Particle size variation of CL@LEV-NPs dispersed in PBS with time extension (*n* = 3).

Under the electron microscope, CL@LEV-NPs showed a spherical shape, uniform size, good dispersion, and no obvious adhesion or local agglomeration (Figures 4A–C) under the electron microscope. Blank nanoparticles are shown in Figure 4B. The nanoparticle size of CL@LEV-NPs was 196.2 ± 2.89 nm, and zeta potential was −14.96 ± 5.35 mV (Figures 4D–E). Detection of sonodynamic properties of LEV was detected and the results showed good ability to produce single linear state oxygen *in vitro* (Figure 4F). The standard curve of CL and LEV aqueous solution was drawn (Supplementary Figures S2B,E) and a linear equation underneath the concentration-absorbance intensity relationship was calculated, as shown in Supplementary Figures S2A,C. The drug loading and encapsulation efficiencies of LEV and CL were (3.8 ± 0.09)%, (3.5 ± 0.12)%, (69.3 ± 0.52)%, and (63.5 ± 0.86)%, respectively (Table 1). The analysis from UV–vis spectrophotometry showed the characteristic absorption peaks of CL@LEV-NPs at 281 nm and 303 nm, respectively, thus proving that the drugs (CL and LEV) were successfully encapsulated into the nanoparticles (Figure 4G). The particle size of CL@LEV-NPs dispersed in PBS did not change significantly within 7 days (Figure 4I), indicating the good stability of CL@LEV-NPs.

**Table 1:** The physical characterization of the nanoparticles.

Formulations	Particle size (nm)	Zeta potential (mV)	PDI	LC (%)	EE (%)
Blank-NPs	162.8 ± 0.49	−13.5 ± 0.61	0.040 ± 0.018	——	——
LEV-NPs	206.2 ± 2.78	−8.19 ± 0.97	0.092 ± 0.052	3.9 ± 0.12	78.9 ± 0.11
CL-NPs	187.6 ± 6.04	−9.5 ± 0.46	0.126 ± 0.029	3.2 ± 0.07	73.5 ± 4.09
CL@LEV-NPs	196.2 ± 2.89	−14.96 ± 5.35	0.122 ± 0.029	3.8 ± 0.09 (LEV)	69.3 ± 0.52 (LEV)
				3.5 ± 0.12 (CL)	63.5 ± 0.86 (CL)

### 3.2. Ultrasound-triggered drug release *in vitro*

CL and LEV releases in CL@LEV-NPs significantly increased after ultrasound irradiation compared with those without sonication, reaching a platform after 60 h (Figure 4H). At 72 h, CL and LEV releases in CL@LEV-NPs after ultrasound irradiation amounted to 66.1 and 65%, respectively, as compared with 44.53 and 43.40%, respectively, in CL@LEV-NPs without sonication, which showed that ultrasonic irradiation can promote the release of drugs from nanoparticles, thus increasing cumulative release.

### 3.3. Antimicrobial susceptibility of LEV and CL to biofilms

Since the biofilm formation is a hallmark of chronic infection, we explored whether cellulose structures could be detected in mature BCG biofilms *in vitro*. We conducted a series of preliminary experiments and have confirmed that cellulose is the main structural component of the biofilm and have determined the mature growth cycle of BCG biofilms (Supplementary Figures S1A–C,E). Of note, after drug sensitivity testing, we derived MIC and MBEC values of 4 and 1,024 μg/mL for LEV on BCG and BCG biofilm, respectively (Figures 3A,B). The MBEC value was 256 times higher than the MIC. Besides, after treating mature biofilms with CL at three different concentrations of 1.024, 0.512, and 0.256 mg/mL, the BCG biofilm biomass values were significantly decreased compared to the controls (*p* < 0.001); while with increasing concentrations of CL, they reduced to 70.6, 66.7, and 66.2%, respectively (Figure 3C). However, the experiment of bactericidal activity showed CL had no bactericidal effect on BCG planktonic bacteria (Figure 3D). Finally, we screened 0.512 mg/mL of CL as *in vitro* and *in vivo* concentration.

### 3.4. Biofilms penetration of BCG biofilms *in vitro*

**Figure 5:**
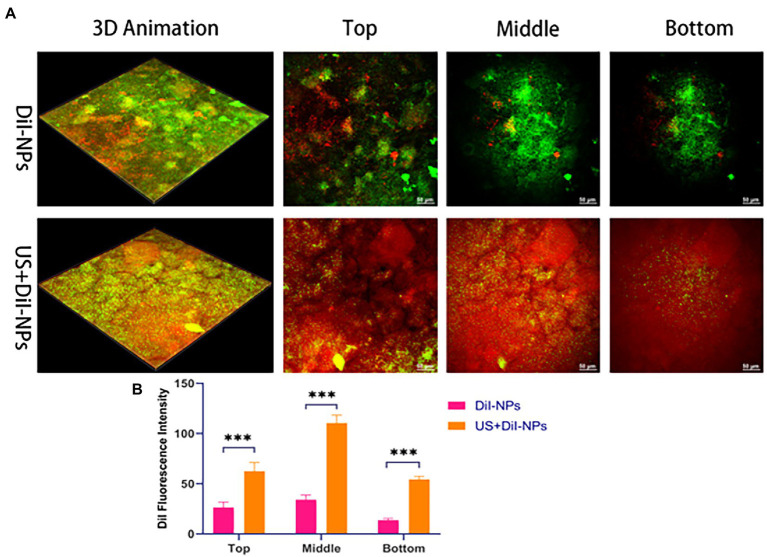
Biofilms permeability analysis following ultrasound treatment. **(A)** CLSM to observe the aggregation of nanoparticles between different layers in the biofilm with and without ultrasonic irradiation, respectively; green fluorescence: SYTO 9 labeled BCG biofilm; red fluorescence: DiI labeled nanoparticles, scale bar 50 μm (200×); **(B)** Quantitative analysis of DiI fluorescence intensity in the top, middle and bottom layer of biofilm after ultrasonic irradiation. ***p < 0.001.

Penetration and distribution of fluorescent nanoparticles DiI-NPs in biofilms were observed by CLSM. As shown in Figure 5A, aggregation of DiI-NPs in different layers of the biofilm significantly increased after ultrasonic irradiation, indicating that ultrasound can effectively promote the entry of nanoparticles into the biofilm. The DiI fluorescence intensity statistics of ultrasonic and non-ultrasonic irradiated nanoparticles in different layers are shown in Figure 5B. The quantitative calculation of fluorescence in each layer of biofilms also showed that the average area of red fluorescence intensity in DiI-NPs ultrasonic group was significantly higher than that in single DiI-NPs group.

### 3.5. *In vitro* BCG biofilms elimination

**Figure 6:**
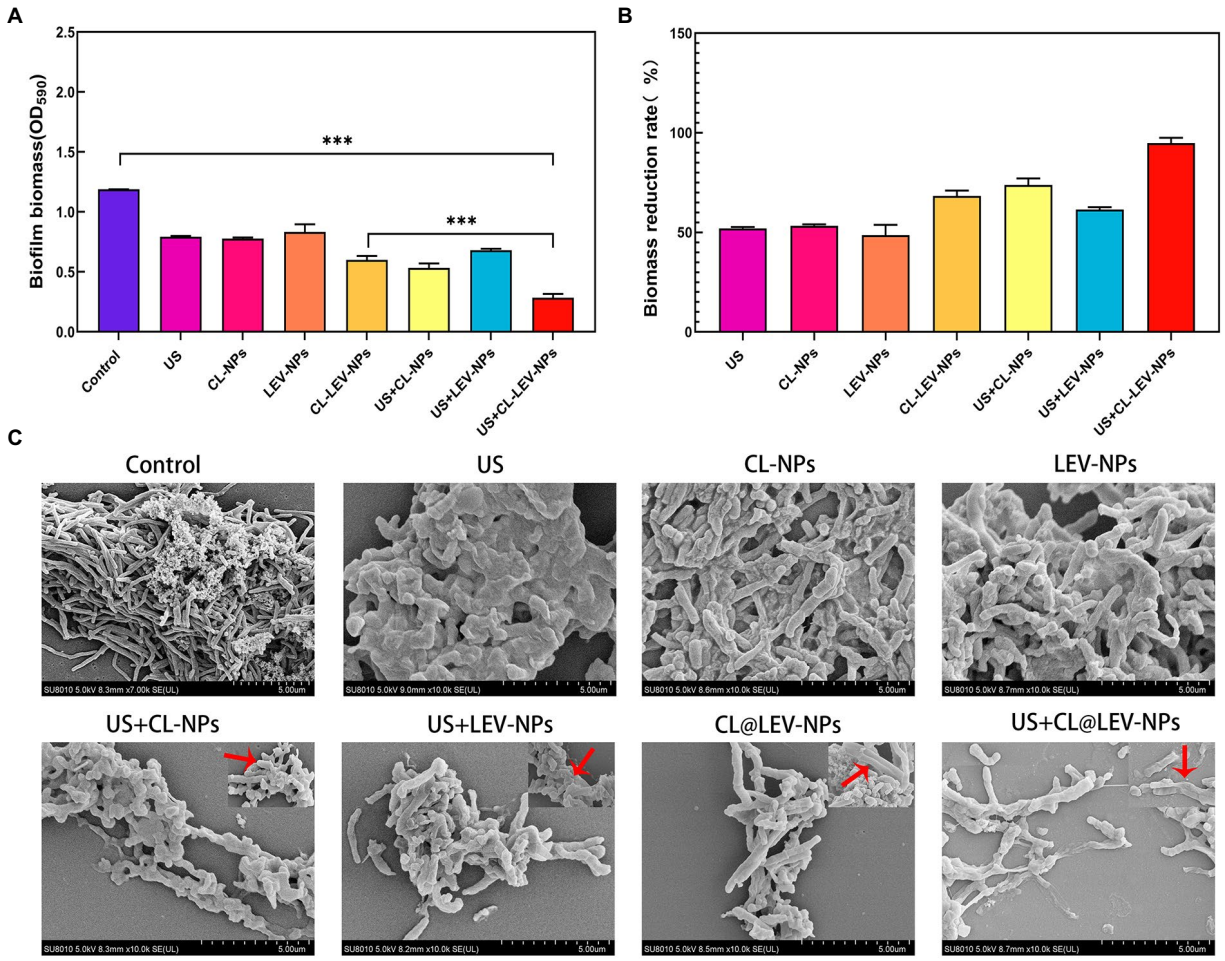
Antibacterial activity of BCG biofilms *in vitro*. **(A)** The quantification of the relative biomass of BCG biofilms after different treatments methods, ***p < 0.001; **(B)** Quantitative analysis of the reduction rate of BCG biofilm after different treatments, biofilm reduction rate = (*OD*_590,Control_−*OD*_590,Test_)/(*OD*_590,Control_) × 100%; **(C)** Typical SEM images of the structural changes of BCG biofilms after different treatments (×10 k). The red arrow indicates the enlarged detail picture (magnificaction,× 20 k).

To quantify biofilm elimination effects of LEV-NPs and CL@LEV-NPs, biofilm biomass value was determined and biofilm reduction rate was calculated using crystal violet assay. The biofilm biomass value in the US + CL@LEV-NPs group was significantly reduced compared to control and single CL@LEV-NPs groups, being the lowest among all the experimental groups (*p* < 0.001) (Figures 6A,B). Moreover, CL@LEV-NPs and CL@LEV-NPs + US could reduce the biomass values of biofilms to 68.3 and 92.8% due to inherent antibacterial and degradable properties of LEV and CL (Figure 6B).

These results were further confirmed by SEM investigation. SEM images demonstrated that the morphology of the BCG biofilm treated with PBS was a three-dimensional structure composed of BCG closely adhering to each other. In sharp contrast, the treatment of CL@LEV-NPs significantly dispersed the BCG biofilm (Figure 6C). Under high magnification, obvious wrinkles, holes, and damages were observed in BCG biofilms exposed to CL@LEV-NPs treatment after US irradiation, as compared with obvious wrinkles on the surface of a small part of bacteria exposed to CL@LEV-NPs treatment without US irradiation. These findings indicate that the CL release from CL@LEV-NPs at 37°C could effectively degrade the BCG biofilm and kill a small part of bacteria, while CL release was accelerated with US irradiation and synergized with US to further eliminate BCG biofilm infections.

### 3.6. Antibacterial activity of BCG biofilms *in vitro*

**Figure 7:**
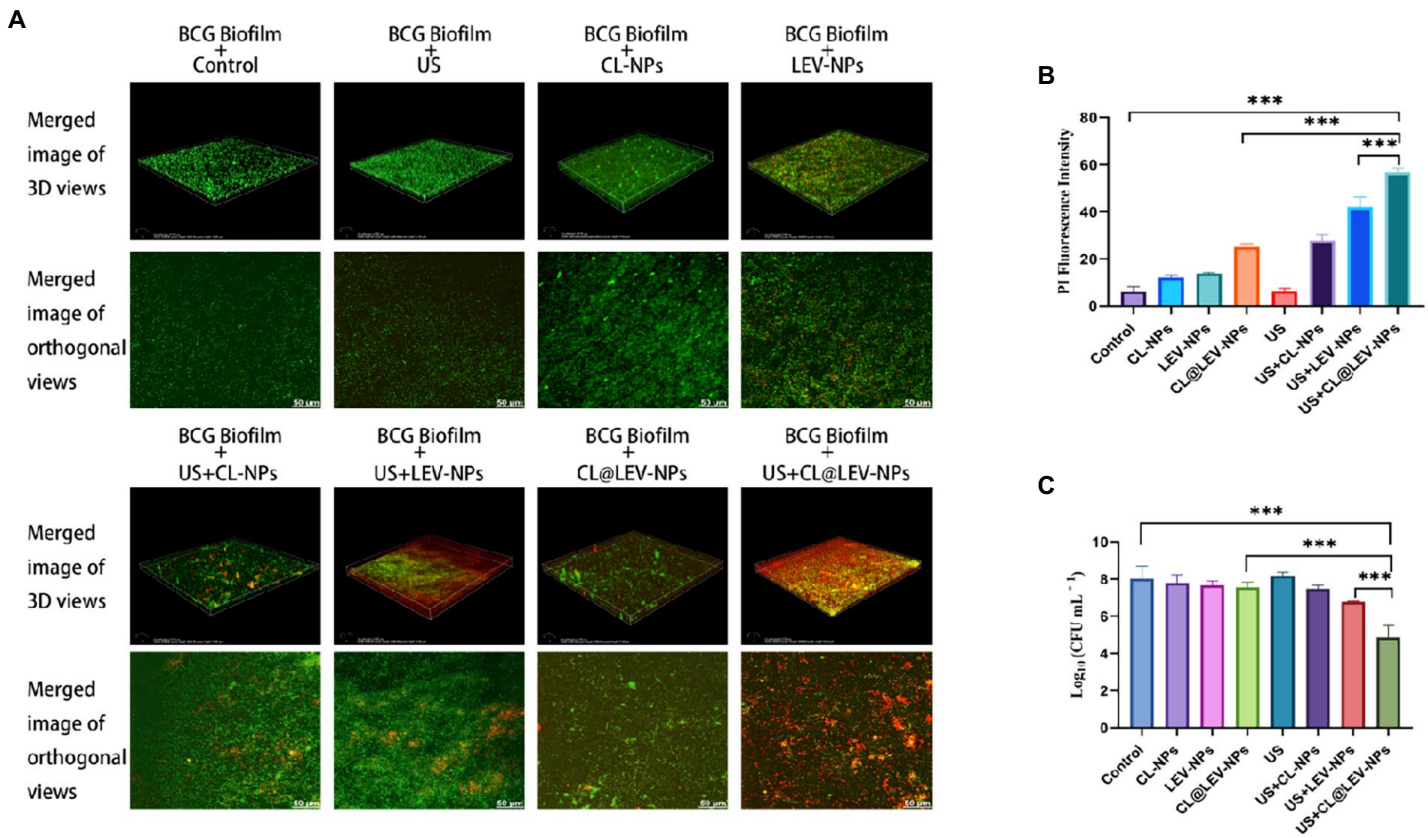
Antibacterial activity of BCG biofilm *in vitro*. **(A)** SYTO 9/PI Staining to observe the 3D reconstruction map and maximum area projection (200×) of BCG biofilm treated by different experimental groups; **(B)** Quantitative analysis of BCG biofilm after different treatments red fluorescence intensity; **(C)** Bacterial plate counts for each group at the end of the different experiments ***p < 0.001.

To further investigate the antibacterial effects of different treatments on the BCG biofilm, we used CLSM to evaluate the BCG biofilm killing effect of LEV-NPs and CL@LEV-NPs with or without US by Live/Dead dyes (SYTO 9 and PI) staining. As shown in Figure 7A, the control group displayed an integrated and dense piece of the membrane with green fluorescence, indicating that the biofilm was not destroyed, while LEV-NPs group displayed a slightly decreased density and thickness but still high activity of BCG biofilm due to the unbroken biofilm integrity. The addition of CL (CL@LEV-NPs group) accelerated the dispersal of the biofilm, which agrees with the result from the crystal violet assay. After 5 min of US irradiation, the CL@LEV-NPs + US group showed the decreased density and thickness of the BCG biofilm and a large number of dead bacteria. The quantitative analysis of red fluorescence intensity is shown in Figure 7B. In combination of US, the anti-biofilm property of CL@LEV-NPs was further strengthened significantly. The reason is that high concentration of CL not only reduced the protective effect of the biofilm but also synergized with US and LEV to achieve more effective killing of BCG under the biofilm protection. Calculating Log10 CFU/mL for bacterium colony counts after 24 h incubation showed that the living colonies in US+CL@LEV-NPs group were significantly decreased compared to all other groups (*p* < 0.001), and US+CL@LEV-NPs exhibited better anti-biofilm properties than US+LEV-NPs without CL (Figure 7C).

### 3.7. Sonodynamic effects of CL@LEV-NPs promote ROS production

**Figure 8:**
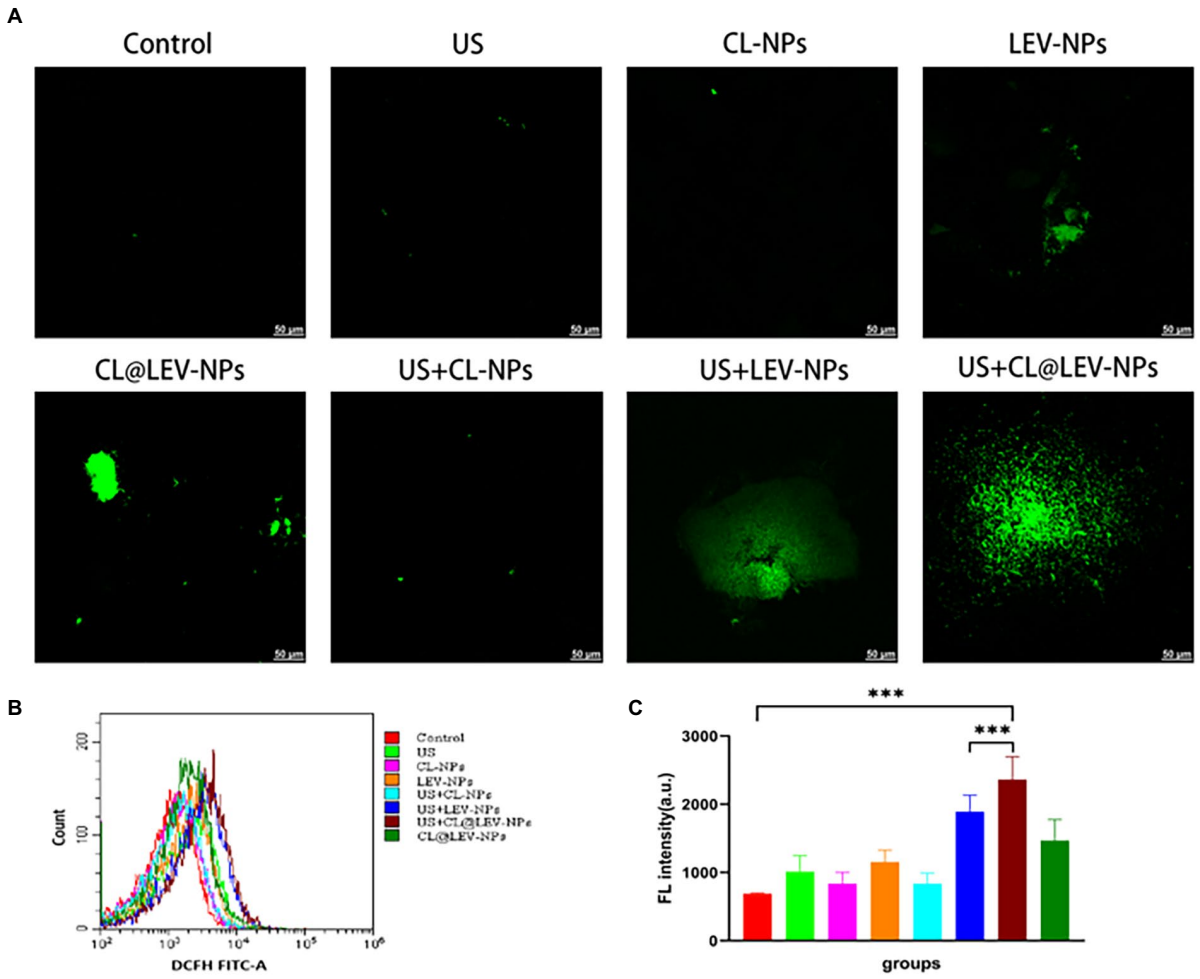
Determination of ROS production. **(A)** CLSM observation of reactive oxygen species in bacteria after different treatment of BCG biofilm *in vitro*, scale bar 50 μm; **(B)** Flow cytometry to detect the mean fluorescence of DCFH-FITC-A after treatment of BCG biofilm in different ways; **(C)** Intensity quantitative analysis of ROS content.

The capability of ultrasound mediated CL @ LEV-NPs to produce ROS in BCG *in vitro* was observed by CLSM and flow cytometry with DCFH-DA (Figure 8A). CLSM qualitative observation found that the ROS levels (green fluorescence) in the US+LEV-NPs group and the US+CL@LEV-NPs group were higher than those in the other groups, with the least green fluorescence signal in the control group and the experimental group without ultrasound excitation (Figure 8A). The quantification results of ROS level by flow cytometry were consistent with the CLSM observation, as shown in Figure 8B. The green fluorescence intensity of the US+CL@LEV-NPs group was significantly higher than that of all the other groups, reaching 2361.5 ± 272.7 (*p* < 0.001) (Figure 8C).

### 3.8. *In vivo* elimination of implant biofilms infections

**Figure 9:**
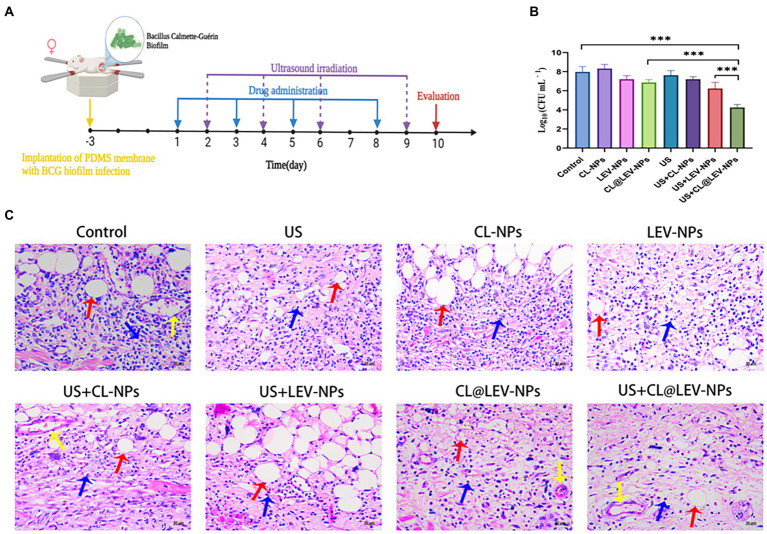
*In vivo* biofilm infection treatment. **(A)** Schematic diagram of the treatment of BCG biofilm infection; **(B)** The bacterial plate count of the surrounding tissue of the mice in each group after the treatment, ***p < 0.001; **(C)** Under different treatment methods HE-stained images of surrounding tissues (blue arrows: inflammatory cells, red arrows: subcutaneous fat, yellow arrows: blood vessels), scale bar 50 μm (200×).

A schematic of the *in vivo* treatment protocol is shown in Figure 9A. To evaluate the anti-infective therapeutic properties of CL@LEV-NPs quantitatively, the PDMS slices and surrounded tissue were removed from mice 2 days after treatment completion. The quantification analysis of bacteria on the implants and surrounded tissue after treatment found that the number of bacterial colonies in the CL@LEV-NPs + US group was the lowest (4.25 ± 0.065) compared to the other infection groups (Figure 9B). Furthermore, the implant surrounded tissues were collected and evaluated with H&E staining. When an infection occurs, neutrophils are attracted to the area of the infected tissue to play a role in inhibiting bacterial proliferation, and they are stained blue to in the infected tissue. As shown in Figure 9C, histological examination found obvious blue areas in the control group, indicating that the surrounded tissues were severely infected. Meanwhile, H&E staining displayed the least inflammatory cell aggregation in the infected tissues of the CL@LEV-NPs + US group, indicating that ultrasound treatment combined with CL@LEV-NPs had the greatest effect on BCG biofilm infection.

### 3.9. ELISA analysis

**Figure 10:**
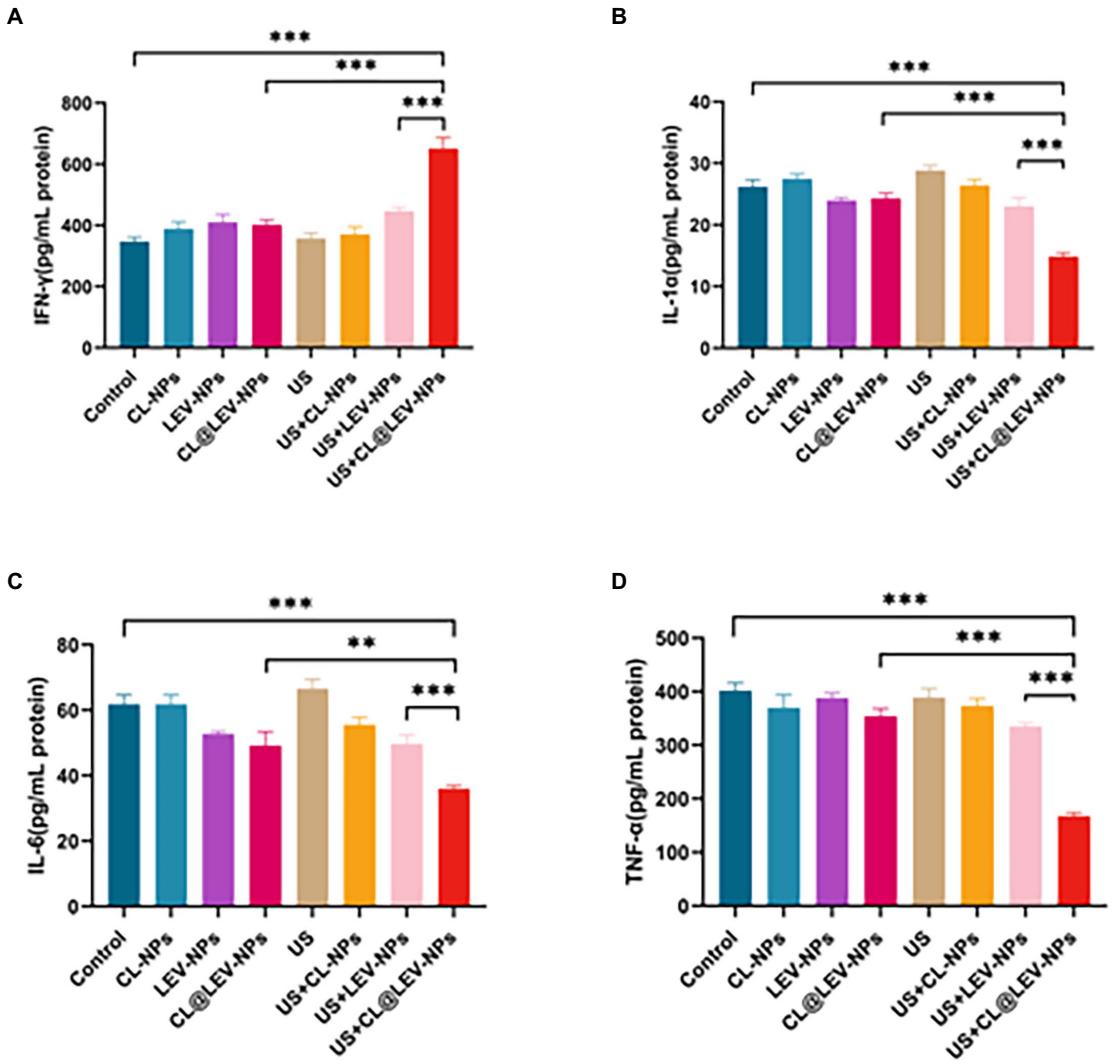
ELISA assay. The concentrations of IFN-γ **(A)**, IL-1α **(B)**, IL-6 **(C),** and TNF-α **(D)** in the serum of BALB/c mice in each group were measured by ELISA after the treatment of different experimental groups,**p < 0.01, ***p < 0.001.

The progression of inflammation is closely related to the level of inflammatory factors. The inflammatory factors associated with bacterial infection of IFN-γ, TNF-α, IL-1α, and IL-6 were measured by ELISA analysis. Serum IFN-γ level in the US+CL@LEV-NPs group was higher than that in the other groups (*p* < 0.001) (Figure 10A), but the TNF-α, IL-1α, and IL-6 levels were lower in the US+CL@LEV-NPs group than in the other groups (*p* < 0.01) (Figures 10B–D).

### 3.10. *In vitro* and *in vivo* biosafety evaluation

**Figure 11:**
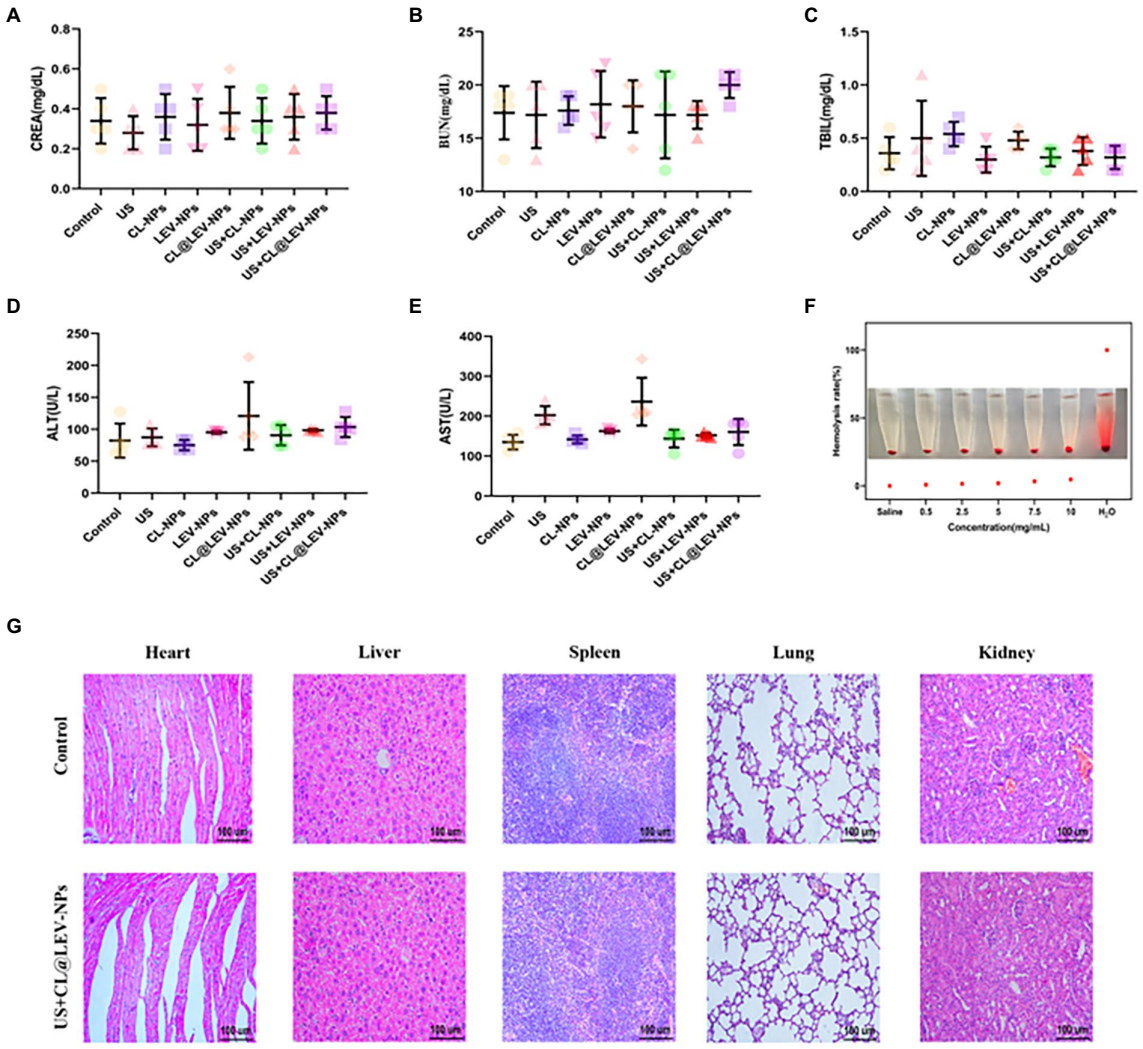
*In vitro* and *in vivo* biosafety evaluation. **(A)** serum CREA, **(B)** BUN, **(C)** TBIL, **(D)** ALT and **(E)** AST indices of BALB/c mice after 12 days of treatment; **(F)** Hemolysis assay; **(G)** H&E staining images of major organs of BALB/c mice after intravenous injection.

To evaluate *in vitro* biocompatibility, we performed *in vitro* blood hemolytic assays and found the hemolysis rate gradually increased with the increase of nanoparticle concentration, but was still lower than 5% even when the concentration increased to 10 mg/mL, indicating that the prepared CL@LEV-NPs had almost no effect on erythrocytes (Figure 11F). To assess the *in vivo* biosafety of CL@LEV-NPs, we observed blood biochemical parameters and compared organ histopathologies in different groups of BALB/c mice. The data of the serum biochemical parameters (ALT, AST, TBIL, CREA, and BUN) were within the normal reference range (Figures 11A–E), indicating that the various treatment methods did not induce liver and kidney function damage. Moreover, after the treatment, we dissected the heart, liver, spleen, lung, and kidney tissues from each group of BALB/c mice and prepared H&E sections. As shown in Figure 11G, BALB/c mice in the US+CL@LEV-NPs group had no significant necrosis, apoptosis, or structural cellular and histological damage in the major organs (heart, liver, spleen, lung, and kidney) compared with those in the control group.

## 4. Discussion

Tuberculosis is a chronic infectious disease with the occurrence of antibiotic recalcitrance, which is often associated with pathogenic biofilm infections (Wang et al., 2017; Martin et al., 2018; Brown, 2021). The biofilm structure as the protective layer for the bacteria is an important reason for the treatment failure of MTB biofilm infections (Liao et al., 2018; Chakraborty et al., 2021). The scientific community has been of interest in new therapeutic approaches against biofilm infections, including targeted degradation of extracellular matrix components to allow more antibiotics to penetrate into the biofilm for effective bactericidal action. In this study, we utilized the combination strategy based on US mediated compound drug-loaded nanoparticles (CL@LEV-NPs) to eliminate the BCG biofilm and kill bacteria inside the biofilm to degrade the structure of biofilm, thus enhance drug penetration in a shorter time and maximize eradication of biofilm.

Cellulose is a key structural component of the extracellular matrix of MTB biofilms and may be speculated as an important target for disrupting the BCG biofilm structure (Trivedi et al., 2016). Previous studies have shown that the combination of CL with anti-tuberculosis drugs INH and RIF could enhance the anti-tuberculosis effect because the main effect of CL is to destroy biofilms rather than *Mycobacterium tuberculosis* cells (Chakraborty et al., 2021). In this study, we first demonstrated that a large number of cellulose structures existed in the BCG biofilm *via* calcofluor white (CW) staining (Supplementary Figure S1) and further treated the BCG biofilm with CL. We found that CL degraded the biofilm in a concentration dependent manner, but did not have a significant antibacterial effect on the free BCG bacterial suspension (Figure 3D).

With the development of efficient drug delivery strategies, it is a new direction to integrate multiple drugs or antibodies into a single nanodrug delivery system (Han et al., 2015; Wan et al., 2020). In this study, the nanoparticles co-loaded with CL and LEV were successfully prepared by the double emulsification method, which are called CL@LEV-NPs with homogeneous in size and regular in shape (Figures 4A,C). In addition, the loading efficiency and encapsulation efficiency of LEV and CL were (3.8 ± 0.09)%, (3.5 ± 0.12)%; (69.3 ± 0.52)%, and (63.5 ± 0.86)%, respectively (Table 1), which is ideal for experimental study of multidrug co-loading in nanoparticles. In addition to effective drug packaging, the efficient site-specific release of the drug at the target site is also a key factor in the design of nanomedical delivery. Under natural conditions, the release rates of CL and LEV of CL@LEV-NPs after 72 h were 44.5 and 43.4%, respectively (Figure 4H). However, when CL@LEV-NPs were combined with US (42 kHz and 0.34 W/cm2) for 5 min, the CL and LEV release rates of CL@LEV-NPs after 72 h reached 66.1 and 65.0%, respectively (Figure 4H). This finding showed that ultrasound irradiation could promote the release of drug from nanoparticles, improve the slow release efficiency, and effectively increase the drug concentration at the local infection site. On the other hand, the nanoparticles themselves could act as exogenous cavitation nuclei, which could enhance the cavitation effect of ultrasound and increase the bacterial biofilm permeability while promoting more drug entry into the biofilm (Yang et al., 2019; Sun et al., 2020; Lyu et al., 2021; Zhang et al., 2021). After ultrasound treatment, the penetration of DiI-NPs (DiI as a model drug, fluoresces red) in the biofilm was observed by CLSM. It was found that ultrasound significantly promoted the penetration of nanoparticles into the bottom of the biofilm (Figure 5), thereby increasing the effective drug concentration in the BCG biofilm.

In this experiment, the antibacterial effect of ultrasound combined with CL@LEV-NPs on BCG biofilm was analyzed from the structure of biofilm to the activity of bacteria hidden in the biofilm. We first verified that the free drug of levofloxacin (LEV) and cellulase (CL) alone exhibited low antibacterial sensitivity to BCG biofilms and had little effect on biofilm clearance (Figure 3D)(Ramage et al., 2001; Naparstek et al., 2014). However, when the two drugs were loaded into PLGA nanoparticles and combined with ultrasound irradiation, the biofilm biomass value in the US+CL@LEV-NPs group was reduced by 92.8%, which was significant compared to control group and single CL@LEV-NPs group(*p* < 0.001) (Figure 6B). Moreover, SEM, as the “gold standard” for measuring the elimination of bacterial biofilms (Miyake et al., 2019) showed that the most serious damages, even the physical damage of bacteria (e.g., fracture) (Figure 6), were observed in US+CL@LEV-NPs group where bacteria were directly exposed to the microenvironment (Figure 6C). In addition, CLSM examination showed a higher proportion of dead bacteria in US+CL@LEV-NPs group than in the other groups (Figure 7A), which confirmed that the combination strategy of ultrasound and CL@LEV-NPs is more effective against BCG biofilm *in vitro* than other treatments.

Next, we further evaluated the therapeutic effect of ultrasound combined with CL@LEV-NPs on biofilm infection *in vivo*. We constructed the BCG biofilm subcutaneous infection model based on PDMS slices. We found that US+CL@LEV-NPs group had the lowest bacterial counts on bacterial plates after treatment (Figure 9B), presumably due to the degradation of the biofilm by CL, allowing for more release of drug into the biofilm by US. Histopathological observations also showed minimal neutrophil aggregation in the tissue structure of infected lesions in US+CL@LEV-NPs group (Figure 9C).

Inflammation manifests itself as vascular response, permeability changes, regenerative repair, etc. (Wei et al., 2015; Das et al., 2016), all of which involve the participation of more cytokines with different functions. The level of cellular inflammatory factors in the animals is also an indirect indicator of the improvement of the bacterial infection (Puntel et al., 2013). The cytokines TNF-α, IL-1α and IL-6 promote the synthesis of acute phase proteins such as C-reactive protein, by hepatocytes, the concentration of which is significantly increased during bacterial infection or tissue damage (Wang et al., 2017). In contrast, IFN-γ can be used to treat chronic granulomas, malignancies and infectious diseases, among others (Bokhari et al., 2008; Shen et al., 2017; Park et al., 2020). After treatment, cytokines in the murine serum from each group were tested by ELISA test (Figure 10). The US+CL@LEV-NPs group had a significantly lower levels of TNF-α, IL-1α, and IL-6 proteins and the higher level of IFN-γ protein than other groups, indicating the combined strategy of ultrasound and CL@LEV-NPs is most effective for inflammation regression.

Another mechanism of enhanced antimicrobial action may be related to sonodynamic antimicrobial chemotherapy effect, which relies on ultrasound activated sonosensitizers of LEV to produce highly oxidative active reactive oxygen species (ROS) and ultimately achieve treatment by eliminating for the removal of bacterial biofilm infections. ROS is the collective name for substances including peroxides, hydroxyl radicals, and superoxide, which can also be effective in the treatment of bacterial infections, and ROS-mediated oxidative stress usually leads to oxidation of biomolecules and cellular components (Sirin and Aslim, 2020), resulting in severe cellular damage. Earlier studies by Gao et al. showed that under the acidic pH conditions of the *Streptococcus pyogenes* biofilm, iron oxide particles could produce strong peroxidase-like activity through local pH-dependent free radicals, leading to extracellular polysaccharide (EPS) degradation and bacterial cell death (Taylor and Webster, 2009). In our experiments, we observed a significant increase in intracellular ROS levels in the ultrasound combined with CL@LEV-NPs group (Figure 8) compared with the control or ultrasound combined with LEV-NPs group, which is consistent with previous research report that LEV can be activated as an acoustic sonosensitizers and generate ROS under ultrasound stimulation (Naumoff, 2011). Besides, the degradation of cellulose by CL resulted in more release of LEV into the BCG biofilm, generating more ROS under the irradiation of ultrasound. Under the synergistic effect of ultrasound, CL and LEV, bacteria were effectively killed and the BCG bacterial biofilm was destroyed (Figure 8C). Overall, the ultrasound treatment platform based on CL@LEV-NPs proposed in this study offered new possibilities for eliminating resistance of TB biofilms.

## 5. Conclusion

CL@LEV-NPs were successfully prepared in this experiment, and a large amount of reactive oxygen species could be generated after ultrasonic irradiation of CL@LEV-NPs. Ultrasound irradiation synergizing with CL@LEV-NPs exhibited significantly removal and bactericidal effects on biofilm by *in vitro* and *in vivo* assays; thus, it can effectively solve the problem of MTB drug resistance. This combination treatment strategy can be applied in the biomedical field and expected for the treatment of chronic infections caused by biofilms.

## Data availability statement

The original contributions presented in the study are included in the article/Supplementary material, further inquiries can be directed to the corresponding authors.

## Ethics statement

The animal study was reviewed and approved by the Animal Ethics Committee of Chongqing Medical University. Written informed consent was obtained from the owners for the participation of their animals in this study.

## Author contributions

All authors listed have made a substantial, direct, and intellectual contribution to the work and approved it for publication.

## Funding

This work was supported by the National Natural Science Foundation of China (No.: 82270115), and Chongqing Graduate Research Innovation Project Fund (No.: CYS21240).

## Conflict of interest

The authors declare that they have no known competing financial interests or personal relationships that could have appeared to influence the work reported in this paper.

## Publisher’s note

All claims expressed in this article are solely those of the authors and do not necessarily represent those of their affiliated organizations, or those of the publisher, the editors and the reviewers. Any product that may be evaluated in this article, or claim that may be made by its manufacturer, is not guaranteed or endorsed by the publisher.

## Abbreviations

MTB: *Mycobacterium tuberculosis*
CL: Cellulase
LEV: Levofloxacin
US: Ultrasound
BCG: *Bacillus Calmette-Guérin*
PLGA: Polylactic acid-glycolic acid
ROS: Reactive oxygen species
TB: Tuberculosis
NIH: National Institutes of Health
EPS: Extracellular polymeric substances
SACT: Sonodynamic antimicrobial chemotherapy
SDT: Sonodynamic therapy
OADC: Oleic acid-albumin-dextrose-catalase
CLSM: Confocal laser scanning microscopy
DiI: 1,1′-dioctadecyl-3,3,3′,3′-tetramethylindo carbocyanine perchlorate
CW: Calcofluor white stain
XTT: Methyl tetrazolium salt
DCFH-DA: 2,7-dichlorodihydrofluorescein diacetate
SOSG: Singlet oxygen sensor green
DL: Loading efficiency
EE: Encapsulation efficiency
OD: Optical density
H&E: Hematoxylin and eosin

## References

[ref1] Alipour M. Suntres Z. E. Omri A. (2009). Importance of DNase and alginate lyase for enhancing free and liposome encapsulated aminoglycoside activity against *Pseudomonas aeruginosa*. J. Antimicrob. Chemother. 64, 317–325. doi: 10.1093/jac/dkp165, PMID: 19465435

[ref3] Bokhari S. M. Kim K. Pinson D. M. Slusser J. Yeh H. W. Parmely M. J. (2008). NK cells and gamma interferon coordinate the formation and function of hepatic granulomas in mice infected with the *Francisella tularensis* live vaccine strain. Infect. Immun. 76, 1379–1389. doi: 10.1128/IAI.00745-07, PMID: 18227174 PMC2292861

[ref4] Brown A. C. (2021). Whole-genome sequencing of mycobacterium tuberculosis directly from sputum samples. Methods Mol. Biol. 2314, 459–480. doi: 10.1007/978-1-0716-1460-0_20, PMID: 34235666

[ref5] Chakraborty P. Bajeli S. Kaushal D. Radotra B. D. Kumar A. (2021). Biofilm formation in the lung contributes to virulence and drug tolerance of mycobacterium tuberculosis. Nat. Commun. 12, 1–17. doi: 10.1038/s41467-021-21748-6 33707445 PMC7952908

[ref6] Chen H. Zhou X. Gao Y. Zheng B. Tang F. Huang J. (2014). Recent progress in development of new sonosensitizers for sonodynamic cancer therapy. Drug Discov. Today 19, 502–509. doi: 10.1016/j.drudis.2014.01.010, PMID: 24486324

[ref7] das M. Sandhu P. Gupta P. Rudrapaul P. de U. C. Tribedi P.. (2016). Attenuation of *Pseudomonas aeruginosa* biofilm formation by Vitexin: a combinatorial study with azithromycin and gentamicin. Sci. Rep. 6, 1–13. doi: 10.1038/srep23347, PMID: 27000525 PMC4802347

[ref8] Flemming H. (2016). EPS-then and now. Microorganisms 4, 1–18. doi: 10.3390/microorganisms4040041 27869702 PMC5192524

[ref9] Flemming H. Neu T. R. Wozniak D. J. (2007). The EPS matrix: the "house of biofilm cells". J. Bacteriol. 189, 7945–7947. doi: 10.1128/JB.00858-07, PMID: 17675377 PMC2168682

[ref10] Floyd K. Glaziou P. Zumla A. Raviglione M. (2018). The global tuberculosis epidemic and progress in care, prevention, and research: an overview in year 3 of the end TB era. Lancet Respir. Med. 6, 299–314. doi: 10.1016/S2213-2600(18)30057-2, PMID: 29595511

[ref11] Getnet F. Demissie M. Worku A. Gobena T. Tschopp R. Farah A. M.. (2021). Challenges in delivery of tuberculosis Services in Ethiopian Pastoralist Settings: clues for reforming service models and organizational structures. BMC Health Serv. Res. 21, 627–641. doi: 10.1186/s12913-021-06662-3, PMID: 34193133 PMC8246683

[ref12] Gutiérrez-Larraínzar M. Rúa J. de Arriaga D. Valle P. García-Armesto M. R. (2013). In vitro assessment of synthetic phenolic antioxidants for inhibition of foodborne *Staphylococcus aureus*, Bacillus cereus and *Pseudomonas fluorescens*. Food Control 30, 393–399. doi: 10.1016/j.foodcont.2012.07.047

[ref13] Han N. Zhao Q. Wan L. Wang Y. Gao Y. Wang P.. (2015). Hybrid lipid-capped mesoporous silica for stimuli-responsive drug release and overcoming multidrug resistance. ACS Appl. Mater. Interfaces 7, 3342–3351. doi: 10.1021/am5082793, PMID: 25584634

[ref14] Hou Y. Yang M. Jiang H. Li D. du Y. (2019). Effects of low-intensity and low-frequency ultrasound combined with tobramycin on biofilms of extended-spectrum beta-lactamases (ESBLs) *Escherichia coli*. FEMS Microbiol. Lett. 366, 1–23. doi: 10.1093/femsle/fnz026 30715289

[ref15] Hu D. Deng Y. Jia F. Jin Q. Ji J. (2020). Surface charge switchable supramolecular nanocarriers for nitric oxide synergistic photodynamic eradication of biofilms. ACS Nano 14, 347–359. doi: 10.1021/acsnano.9b05493, PMID: 31887012

[ref16] Hytonen M. Vanhanen M. Keskinen H. Tuomi T. Tupasela O. Nordman H. (1994). Pharyngeal edema caused by occupational exposure to cellulase enzyme. Allergy 49, 782–784. doi: 10.1111/j.1398-9995.1994.tb02103.x, PMID: 7695070

[ref2] Jean B. Franklin R. Jeff A. Patricia A. George M. Dwight J.. (2021). Performance standards for antimicrobial susceptibility testing. CLSI. 1, 1–230.

[ref17] Kamali E. Jamali A. Izanloo A. Ardebili A. (2021). In vitro activities of cellulase and ceftazidime, alone and in combination against Pseudomonas aeruginosa biofilms. BMC Microbiol. 21, 347–357. doi: 10.1186/s12866-021-02411-y, PMID: 34915848 PMC8675527

[ref18] Kasaeva T. (2022). Global Tuberculosis Report 2022. Geneva: World Health Organization, 1–68.

[ref19] Koh W. Lee S. H. Kang Y. A. Lee C. H. Choi J. C. Lee J. H.. (2013). Comparison of levofloxacin versus moxifloxacin for multidrug-resistant tuberculosis[J]. Am. J. Respir. Crit. Care Med. 188, 858–864. doi: 10.1164/rccm.201303-0604OC, PMID: 23927582

[ref20] Lai Y. Lu N. Ouyang A. Zhang Q. Zhang P. (2022). Ferroptosis promotes sonodynamic therapy: a platinum(II)-indocyanine sonosensitizer. Chem. Sci. 13, 9921–9926. doi: 10.1039/D2SC02597C, PMID: 36128230 PMC9430585

[ref21] Li G. Li J. Hou Y. Xie S. Xu J. Yang M.. (2021). Levofloxacin-loaded Nanosonosensitizer as a highly efficient therapy for bacillus Calmette-Guerin infections based on bacteria-specific labeling and Sonotheranostic strategy. Int. J. Nanomed. 16, 6553–6573. doi: 10.2147/IJN.S321631, PMID: 34602818 PMC8478796

[ref22] Liao A. H. Chuang H. C. Chang B. Y. Kuo W. C. Wang C. H. Gao H. W.. (2018). Combining microbubble contrast agent with pulsed-laser irradiation for transdermal drug delivery. Pharmaceutics 10, 1–14. doi: 10.3390/pharmaceutics10040175 PMC6321619 30282960

[ref23] Limoli D. H. Jones C. J. Wozniak D. J. (2015). Bacterial extracellular polysaccharides in biofilm formation and function.[J]. Microbiol. Spectr. 3, 1–19. 10.1128/microbiolspec.MB-0011-2014 PMC4657554 26185074

[ref24] Liu B. Wang D. Liu B. Wang X. He L. L. Wang J.. (2011). The influence of ultrasound on the fluoroquinolones antibacterial activity[J]. Ultrason. Sonochem. 18, 1052–1056. doi: 10.1016/j.ultsonch.2011.02.001, PMID: 21353619

[ref25] Liu B. Wang J. Wang X. Liu B. M. Kong Y. M. Wang D.. (2010). Spectrometric studies on the Sonodynamic damage of protein in the presence of levofloxacin. J. Fluoresc. 20, 985–992. doi: 10.1007/s10895-010-0645-x, PMID: 20358282

[ref26] Losada E. Hinojosa M. Moneo I. Dominguez J. Gomez M. Ibanez M. (1986). Occupational asthma caused by cellulase. J. Allergy Clin. Immunol. 77, 635–639. doi: 10.1016/0091-6749(86)90358-1, PMID: 3958392

[ref27] Lyu X. Li C. Zhang J. Wang L. Jiang Q. Shui Y.. (2021). A novel small molecule, LCG-N25, inhibits Oral streptococcal biofilm. Front. Microbiol. 12, 1–11. doi: 10.3389/fmicb.2021.654692 PMC8044806 33868212

[ref28] Martin S. E. Nguyen C. M. Basaraba R. J. Melander C. (2018). Analogue synthesis reveals decoupling of antibiofilm and β-lactam potentiation activities of a lead 2-aminoimidazole adjuvant against mycobacterium smegmatis. Chem. Biol. Drug Des. 92, 1403–1408. doi: 10.1111/cbdd.13208, PMID: 29663670 PMC6097888

[ref29] Martins Y. A. Fonseca M. J. V. Pavan T. Z. Lopez R. F. V. (2020). Bifunctional therapeutic application of low-frequency ultrasound associated with zinc Phthalocyanine-loaded micelles. Int. J. Nanomedicine 15, 8075–8095. doi: 10.2147/IJN.S264528, PMID: 33116519 PMC7586016

[ref30] Maurya S. K. Aqdas M. das D. Singh S. Nadeem S. Kaur G.. (2020). A multiple T cell epitope comprising DNA vaccine boosts the protective efficacy of bacillus Calmette-Guerin (BCG) against mycobacterium tuberculosis. BMC Infect. Dis. 20, 1–14. doi: 10.1186/s12879-020-05372-1, PMID: 32942991 PMC7495405

[ref31] Miyake H. Miyazaki D. Shimizu Y. Sasaki S. I. Baba T. Inoue Y.. (2019). Toxicities of and inflammatory responses to moxifloxacin, cefuroxime, and vancomycin on retinal vascular cells. Sci. Rep. 9, 1–11. doi: 10.1038/s41598-019-46236-2 31278356 PMC6611880

[ref32] Naparstek L. Carmeli Y. Navon-Venezia S. Banin E. (2014). Biofilm formation and susceptibility to gentamicin and colistin of extremely drug-resistant KPC-producing Klebsiella pneumoniae. J. Antimicrob. Chemother. 69, 1027–1034. doi: 10.1093/jac/dkt487, PMID: 24408988

[ref33] Naumoff D. G. (2011). Hierarchical classification of glycoside hydrolases[J]. Biochem. Moscow 76, 622–635. doi: 10.1134/S0006297911060022, PMID: 21639842

[ref34] Park H. Park H. Shim S. Kim S. Shin M. K. Yoo H. S. (2020). Epithelial processed Mycobacterium avium subsp. paratuberculosis induced prolonged Th17 response and suppression of phagocytic maturation in bovine peripheral blood mononuclear cells. Sci. Rep. 10, 1–11. doi: 10.1038/s41598-020-78113-8, PMID: 33273606 PMC7713309

[ref35] Puntel G. O. Carvalho N. R. Dobrachinski F. Salgueiro A. C. F. Puntel R. L. Folmer V.. (2013). Cryotherapy reduces skeletal muscle damage after ischemia/reperfusion in rats. J. Anat. 222, 223–230. doi: 10.1111/joa.12009, PMID: 23231035 PMC3632227

[ref36] Ramage G. Vande Walle K. Wickes B. L. López-Ribot J. ´. L. (2001). Standardized method for in vitro antifungal susceptibility testing of Candida albicans biofilms. Antimicrob. Agents Chemother. 45, 2475–2479. doi: 10.1128/AAC.45.9.2475-2479.2001, PMID: 11502517 PMC90680

[ref37] Salgaonkar V. A. Datta S. Holland C. K. Mast T. D. (2009). Passive cavitation imaging with ultrasound arrays. J. Acoust. Soc. Am. 126, 3071–3083. doi: 10.1121/1.3238260, PMID: 20000921 PMC2803721

[ref38] Shen Y. Hu W. Wei Y. Feng Z. Yang Q. (2017). The immune mechanism of mycoplasma hyopneumoniae 168 vaccine strain through dendritic cells. BMC Vet. Res. 13, 1–7. doi: 10.1186/s12917-017-1194-1 28915878 PMC5603027

[ref39] Sirin S. Aslim B. (2020). Characterization of lactic acid bacteria derived exopolysaccharides for use as a defined neuroprotective agent against amyloid beta(1-42)-induced apoptosis in SH-SY5Y cells. Sci. Rep. 10, 1–18. doi: 10.1038/s41598-020-65147-1 32415207 PMC7229009

[ref40] Suk J. S. Xu Q. Kim N. Hanes J. Ensign L. M. (2016). PEGylation as a strategy for improving nanoparticle-based drug and gene delivery. Adv. Drug Deliv. Rev. 99, 28–51. doi: 10.1016/j.addr.2015.09.012, PMID: 26456916 PMC4798869

[ref41] Sun D. Pang X. Cheng Y. Ming J. Xiang S. Zhang C.. (2020). Ultrasound-switchable nanozyme augments sonodynamic therapy against multidrug-resistant bacterial infection. ACS Nano 14, 2063–2076. doi: 10.1021/acsnano.9b08667, PMID: 32022535

[ref42] Tang Y. Wang T. Feng J. Rong F. Wang K. Li P.. (2021). Photoactivatable nitric oxide-releasing gold Nanocages for enhanced hyperthermia treatment of biofilm-associated infections. ACS Appl. Mater. Interfaces 13, 50668–50681. doi: 10.1021/acsami.1c12483, PMID: 34669372

[ref43] Taylor E. N. Webster T. J. (2009). The use of superparamagnetic nanoparticles for prosthetic biofilm prevention. Int. J. Nanomedicine 4, 145–152. doi: 10.2147/IJN.S5976, PMID: 19774113 PMC2747349

[ref44] Trivedi A. Mavi P. S. Bhatt D. Kumar A. (2016). Thiol reductive stress induces cellulose-anchored biofilm formation in mycobacterium tuberculosis. Nat. Commun. 7, 1–15. doi: 10.1038/ncomms11392 PMC4848537 27109928

[ref45] van Wyk N. Navarro D. Blaise M. Berrin J. G. Henrissat B. Drancourt M.. (2017). Characterization of a mycobacterial cellulase and its impact on biofilm-and drug-induced cellulose production. Glycobiology 27, 392–399. doi: 10.1093/glycob/cwx014, PMID: 28168306

[ref46] Wan Y. Xu W. Ren X. Wang Y. Dong B. Wang L. (2020). Microporous frameworks as promising platforms for antibacterial strategies against oral diseases. Front. Bioeng. Biotechnol. 8, 1–20. doi: 10.3389/fbioe.2020.00628 32596233 PMC7304413

[ref47] Wang D. Cheng D. Ji L. Niu L. J. Zhang X. H. Cong Y.. (2021). Precise magnetic resonance imaging-guided sonodynamic therapy for drug-resistant bacterial deep infection. Biomaterials 264, 120386–120311. doi: 10.1016/j.biomaterials.2020.120386, PMID: 32979656

[ref48] Wang W. Shao Y. Li S. Xin N. Ma T. Zhao C.. (2017). Caspase-11 plays a protective role in pulmonary *Acinetobacter baumannii* infection. Infect. Immun. 85, 1–18. doi: 10.1128/IAI.00350-17 PMC5607408 28760936

[ref49] Wang H. Teng F. Yang X. Guo X. Tu J. Zhang C.. (2017). Preventing microbial biofilms on catheter tubes using ultrasonic guided waves. Sci. Rep. 7, 616–635. doi: 10.1038/s41598-017-00705-8, PMID: 28377583 PMC5429618

[ref50] Wei X. L. Yin J. Zou G. Y. Zhang Z. T. Walley J. Harwell J.. (2015). Treatment interruption and directly observed treatment of multidrug-resistant tuberculosis patients in China. Int. J. Tuberc. Lung Dis. 19, 413–419. doi: 10.5588/ijtld.14.0485, PMID: 25859996

[ref51] Wolfmeier H. Pletzer D. Mansour S. C. Hancock R. E. W. (2018). New perspectives in biofilm eradication. ACS Infect. Dis. 4, 93–106. doi: 10.1021/acsinfecdis.7b00170 29280609

[ref52] Xie S. Li G. Hou Y. Yang M. Li F. Li J.. (2020). A synergistic bactericidal effect of low-frequency and low-intensity ultrasound combined with levofloxacin-loaded PLGA nanoparticles onM. Smegmatisin macrophages. J. Nanobiotechnol. 18, 107–122. doi: 10.1186/s12951-020-00658-7, PMID: 32727616 PMC7388535

[ref53] Yang M. Du K. Hou Y.. (2019). Synergistic antifungal effect of amphotericin B-loaded poly(lactic-co-glycolic acid) nanoparticles and ultrasound against Candida albicans biofilms. Antimicrob. Agents Chemother. 63, 1–13. doi: 10.1128/AAC.02022-18 PMC6437511 30670414

[ref54] Zhang Z. Li Y. Zhang T. Shi M. Song X. Yang S.. (2021). Hepatocyte growth factor-induced tendon stem cell conditioned medium promotes healing of injured Achilles tendon[J]. Front. Cell Dev. Biol. 9, 1–13. doi: 10.3389/fcell.2021.654084 PMC8059769 33898452

[ref55] Zuo X. Gao H. Gao M. Jin Z. Tang Y. Z. (2021). Antibacterial activity of a promising antibacterial agent: 22-(4-(2-(4-Nitrophenyl-piperazin-1-yl)-acetyl)-piperazin-1-yl)-22-deoxypleuromutilin. Molecules 26, 1–13. doi: 10.3390/molecules26123502, PMID: 34201372 PMC8227856

