# Supplementary material for: Synergistic antibacterial effects of ultrasound combined nanoparticles encapsulated with cellulase and levofloxacin on Bacillus Calmette-Guérin biofilms
Source: Front Microbiol. 2023 Mar 1;14:1108064. doi: 10.3389/fmicb.2023.1108064 (PMC10014853; doi:10.3389/fmicb.2023.1108064)
Supplement: Supplementary file 1 [file Data_Sheet_1.docx]

Supporting Information

1. **Supplementary Data**

## Establishment of BCG Culture and Biofilms Model

The bacteria were incubated in a 37°C shaker incubator at 200 r/min until the OD600nm of the BCG broth (10% OADC booster, 0.2% glycerol and 0.5% Tween-80) was 1.0. The bottom BCG bacteria were collected by centrifugation at 4000 rpm for 10 min and resuspended in Tween-free 7H9 medium. Then the diluted BCG bacterial solution was added into 96-well plates and 35 mm Petri dishes (2 mL bacterial solution per dish) respectively. Finally, they were placed in an incubator at 37°C for static incubation, and 100 μL of XTT solution (20% XTT + 1% menaquinone + 79% PBS) was added to the 96-well plates every 24 h. After the reaction at 37°C and protected from light for 3 h, the metabolic activity of BCG biofilm growth was indicated by measuring the change of color in the solution at 490 nm using an enzyme marker.The biofilm maturation growth cycle of BCG was determined by removing a Petri dish every 24 h for staining with crystal violet (0.1%) and observing the morphological and structural changes of the biofilm under a light microscope.

## Characteristic Absorption Peaks of Drugs and Standard Curve Plotting

Weighed the appropriate amount of cellulase and levofloxacin, dissolved with DMSO, and then quantitatively diluted with double-distilled water into a solution with a concentration of 5 μg/mL, and scanned the UV absorption spectrum in the wavelength range of 200-400 nm using double-distilled water as blank.Weigh 10 mg of levofloxacin, dissolve it with a small amount of methanol, transfer it into a 10 mL volumetric flask, fix the volume with double-distilled water to make a reserve solution with a concentration of 1 mg/mL and store it at 4℃. The reserve solution was diluted and fixed with double-distilled water to make a series of standard solutions of 2, 3.5, 5, 6.5, 8, 9.5, 11 and 12.5 μg/mL. The absorption values were measured at the maximum absorption wavelength with double-distilled water as blank control, and the absorption value A was recorded.The same steps for the standard curve of cellulase.

## Safety Validation of Ultrasound Percutaneous Irradiation in Mice

To confirm the safe dose of ultrasound percutaneous irradiation used, BALB/c were irradiated with different intensities (0.3, 0.5 and 0.63 W/cm2) and duration (5, 10 and 15 min) of ultrasound to observe skin damage after removal of hair from the dorsal skin. After 24 h of ultrasound treatment, skin biopsies of the irradiated areas were taken for histopathological observation by cutting 5.0 μm thick sections and staining with H&E to observe the skin tissue damage under a light microscope.

## Modeling Biofilms Infection *in Vivo*

Polydimethylsiloxane (PDMS) slices (0.7×0.4 cm) were completely immersed in 3 mL of 7H9 medium containing BCG (108 CFU/ml) and cultured for 10d (37 ± 0.5℃) to form biofilms on the surface of the PDMS slices. The PDMS slices were then surgically implanted into the back of the mice (25-30 g). The biofilm infected PDMS slices were sandwiched between the muscle and the epidermis, separated from the muscle by the peritoneum. After 3 days implantation , some of their tissues were taken to confirm BCG biofilm infection by anti-acid staining.

1. **Supplementary Figures**


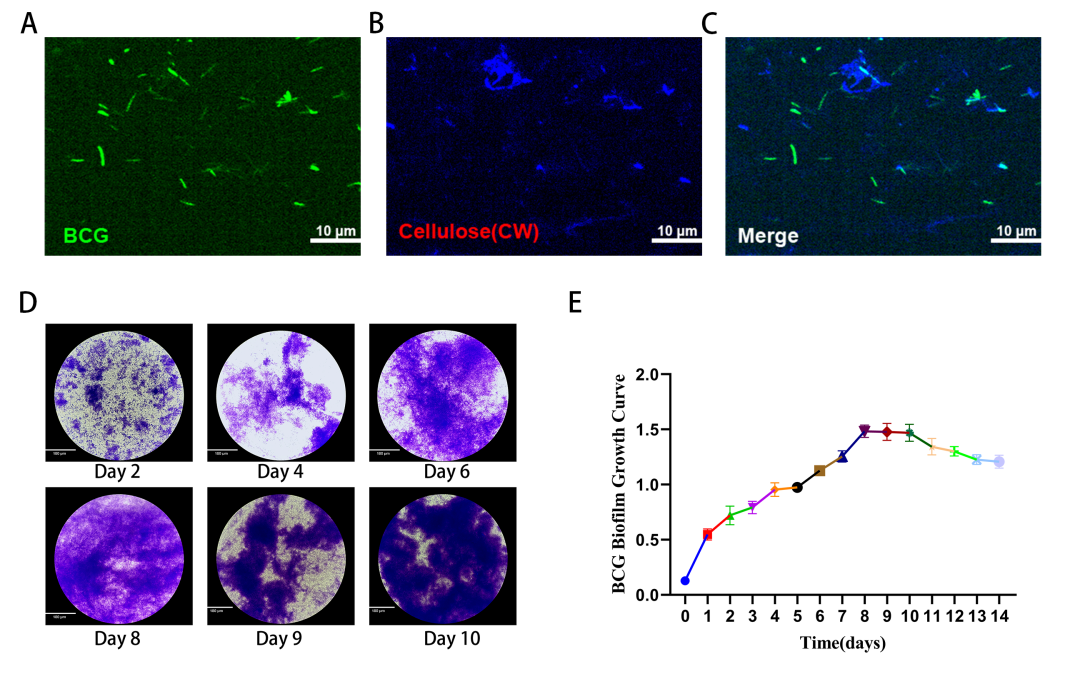


**Fig. S1** BCG forms containing cellulose biofilms in vitro & BCG biofilm maturation cycle (A-C) SYTO 9 (green) staining and CW (blue) staining of the BCG suggesting encapsulation of BCG within a cellulose-rich extracellular matrix (magnification,×400); (D) Observation of BCG biofilm formation process by 0.1% crystal violet staining (magnification, ×200); (E) Growth kinetic curve of BCG biofilm by XTT method.


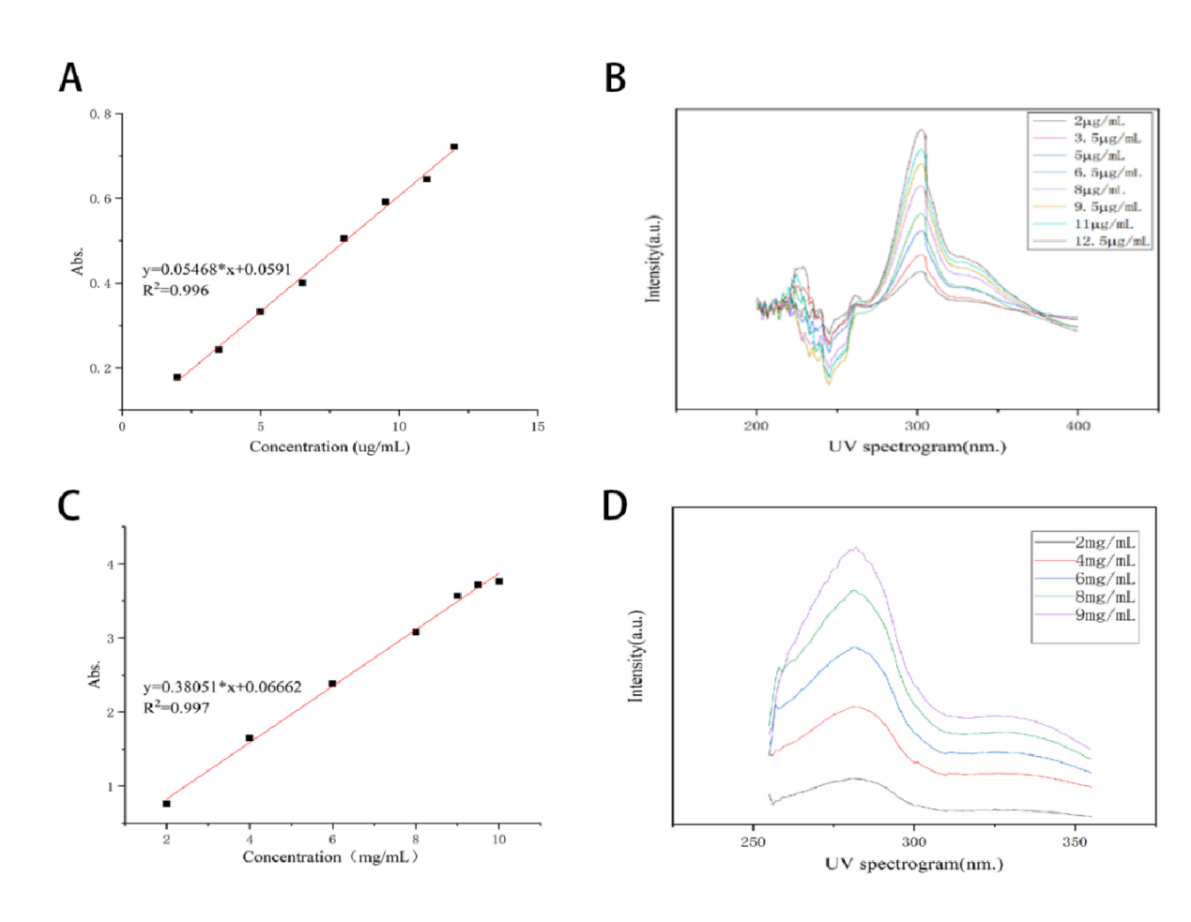


**Fig. S2** Basic characteristics of nanoparticles (A) UV spectra of different concentrations of LEV; (B) Standard curve of LEV at different concentrations of free at the maximum absorption peak (303nm); (C) UV spectra of different concentrations of CL; (D) Standard curve of CL at different concentrations of free at the maximum absorption peak (281 nm).


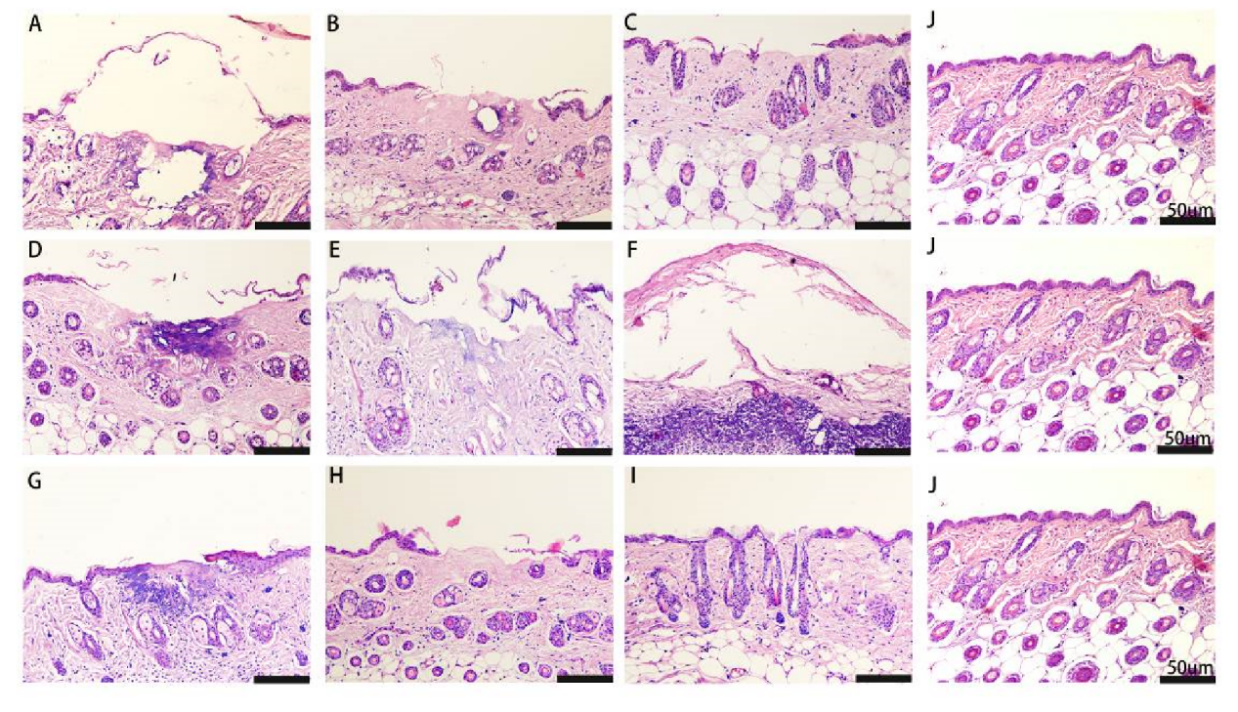


**Fig.S3** Safety validation of low-frequency, low-intensity ultrasound percutaneous irradiation in mice (A. B. &C) Modeled BALB/c mouse skin after 15 min, 10 min, 5 min of irradiation power 0.63 W/cm2; (D. E. & F) Modeled BALB/c mouse skin after 15 min, 10 min, 5 min of irradiation power 0.5 W/cm^2^; ( G. H. & I) Modeled BALB/c mouse skin after 15 min, 10 min, 5 min of irradiation power 0.34 W/cm^2^ and 5 min; J. Modeled BALB/c mouse skin without ultrasound irradiation (magnification, ×200).


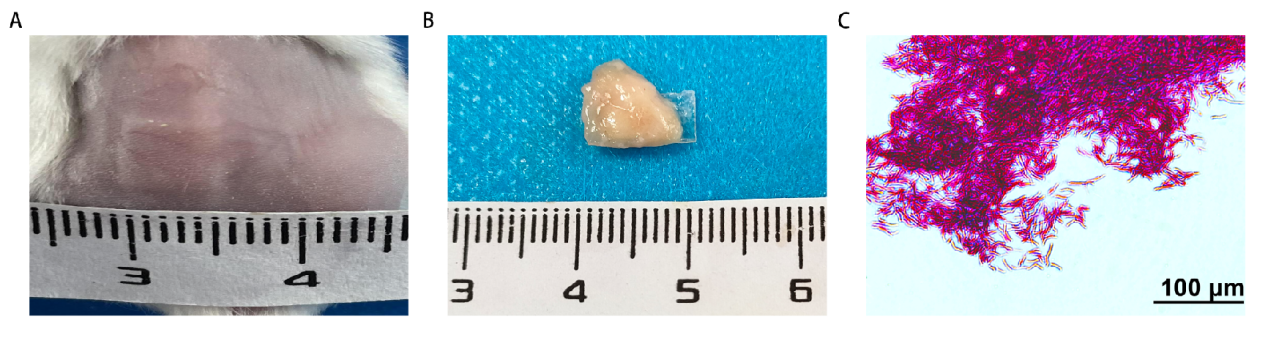


**Fig.S4** Modeling biofilm infection in vivo (A) BALB/c mice were implanted subcutaneously with infected biofilm membrane to establish an in vivo model; (B) The BCG-infected PDMS sections were implanted subcutaneously in mice for 3 days after taking the actual images; (C) Light micrograph of acid-fast staining of a portion of the sample on PDMS section (amplification,×1000).
